# Surface-active antibiotic production as a multifunctional adaptation for postfire microorganisms

**DOI:** 10.1093/ismejo/wrae022

**Published:** 2024-02-05

**Authors:** Mira D Liu, Yongle Du, Sara K Koupaei, Nicole R Kim, Monika S Fischer, Wenjun Zhang, Matthew F Traxler

**Affiliations:** Department of Chemistry, University of California, Berkeley, CA 94720, United States; Department of Chemical and Biomolecular Engineering, University of California, Berkeley, CA 94720, United States; Department of Plant and Microbial Biology, University of California, Berkeley, CA 94720, United States; Department of Plant and Microbial Biology, University of California, Berkeley, CA 94720, United States; Department of Plant and Microbial Biology, University of California, Berkeley, CA 94720, United States; Department of Chemical and Biomolecular Engineering, University of California, Berkeley, CA 94720, United States; Department of Plant and Microbial Biology, University of California, Berkeley, CA 94720, United States

**Keywords:** fire, antibiotics, surfactants, interspecies interactions, motility

## Abstract

Wildfires affect soils in multiple ways, leading to numerous challenges for colonizing microorganisms. Although it is thought that fire-adapted microorganisms lie at the forefront of postfire ecosystem recovery, the specific strategies that these organisms use to thrive in burned soils remain largely unknown. Through bioactivity screening of bacterial isolates from burned soils, we discovered that several *Paraburkholderia* spp. isolates produced a set of unusual rhamnolipid surfactants with a natural methyl ester modification. These rhamnolipid methyl esters (RLMEs) exhibited enhanced antimicrobial activity against other postfire microbial isolates, including pyrophilous *Pyronema* fungi and *Amycolatopsis* bacteria, compared to the typical rhamnolipids made by organisms such as *Pseudomonas* spp. RLMEs also showed enhanced surfactant properties and facilitated bacterial motility on agar surfaces. *In vitro* assays further demonstrated that RLMEs improved aqueous solubilization of polycyclic aromatic hydrocarbons, which are potential carbon sources found in char. Identification of the rhamnolipid biosynthesis genes in the postfire isolate, *Paraburkholderia kirstenboschensis* str. F3, led to the discovery of *rhlM*, whose gene product is responsible for the unique methylation of rhamnolipid substrates. RhlM is the first characterized bacterial representative of a large class of integral membrane methyltransferases that are widespread in bacteria. These results indicate multiple roles for RLMEs in the postfire lifestyle of *Paraburkholderia* isolates, including enhanced dispersal, solubilization of potential nutrients, and inhibition of competitors. Our findings shed new light on the chemical adaptations that bacteria employ to navigate, grow, and outcompete other soil community members in postfire environments.

## Introduction

Wildfires represent an archetypal disturbance regime that affects communities of animals, plants, and microorganisms. The below-ground effects of fire on the soil nutrient landscape are stratified, with the most intense alterations occurring at the surface. In the top layer of soil, fire reduces the amount of bioavailable carbon, as extreme heat leads to combustive release of carbon dioxide, and most of the remaining carbon is converted into pyrolyzed organic matter (PyOM) [1, 2]. PyOM predominantly consists of stable, aromatic C products including polycyclic aromatic hydrocarbons (PAHs). These recalcitrant molecules are not only difficult to degrade, but are also hydrophobic and poorly bioavailable.

Organisms from fire-prone ecosystems often possess adaptations that enable survival or rapid recolonization after fire. For example, many conifers produce thick protective bark and fire-activated serotinous cones [3]. Previous research suggests that soil microorganisms lie at the forefront of postfire community recovery processes [4]. Unlike most plants and animals, some bacteria and fungi can access pyrolyzed chemical products and reintegrate them into the local food web, as in the case of fungi such as *Pyronema* spp. [5, 6]. However, microorganisms face numerous other challenges in a burned soil environment beyond nutrient limitation, such as poor nutrient solubility, increased hydrophobicity of the local environment, and competition with other fire-adapted organisms. Thus, PyOM-adapted catabolism alone may be an insufficient strategy to thrive given the multifaceted demands presented by the postfire environment.

After a perturbation such as fire, organismal communities can recover over time through the process of ecological succession. In perturbed systems with limited or altered nutrient availability, competitive interactions between colonizing members may play an important role in shaping the developing community. Early microbial colonizers, such as certain species of fungi, are termed “pyrophilous” because they consistently emerge after fire. The fungal genus *Pyronema* contains multiple pyrophilous species that often rapidly dominate postfire microbial communities [7]. However, after a short-lived peak, these *Pyronema* species sharply decline in abundance. Numerous factors may be responsible for the decline of *Pyronema*, such as nutrient depletion or changes in pH. Alternatively, the rapid decline of *Pyronema* might result from competitive interspecies interactions.

In this work, we sought to explore the possibility that interference competition mediated by specialized metabolism, including the production of inhibitory secondary metabolites, could play a role in postfire microbial interactions. We also considered whether such specialized metabolites (SMs) might confer other adaptive advantages within postfire environments. Recent work has underscored the notion that SMs may have multiple functions in nutrient limited environments. For instance, microbially produced phenazines have long been recognized as antimicrobial agents, but recently have been shown to play roles in enhancing the bioavailability of phosphorus [8] and iron [9], and in redox balancing under anaerobic conditions [10]. Given the unique challenges present in postfire soils, SMs that possess specific physicochemical properties may be particularly relevant in this natural context. For example, molecules that are surface-active may impart advantages regarding the hydrophobicity of burned soils, by promoting motility and/or enhancing solubility of pyrolyzed carbon sources. Thus, we hypothesized that adaptations of postfire microorganisms may present new opportunities for (1) discovery of novel specialized metabolites, and (2) understanding the roles of specialized metabolites *in situ.*

To explore these possibilities, we screened for molecules produced by bacteria isolated from postfire soils that could inhibit the growth of *Pyronema*. We report that multiple postfire isolates of the genus *Paraburkholderia* produced novel members of a class of biosurfactants, rhamnolipid methyl esters (RLMEs), which likely provide multiple adaptive advantages for their producers. Collectively, our findings shed light on chemical adaptations that bacteria may employ in burned soil environments in order to grow, survive, and outcompete other community members. Beyond this, our results highlight microorganisms from perturbed environments as fruitful sources for discovery of novel compounds and enzymes.

## Results

### Screening burned soil bacterial isolates for activity against *Pyronema omphalodes*

**Figure 1:**
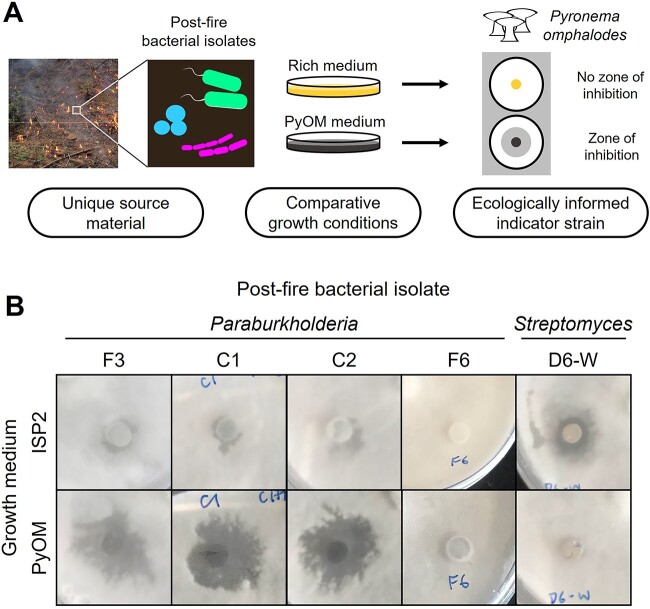
(A) Ecologically based natural product discovery framework; bacterial isolates from postfire soils were cultivated in parallel on a rich medium and PyOM-containing medium; solid cultures were screened using an agar plug assay for antifungal activity against *P. omphalodes*; (B) preliminary antifungal screening results for select postfire bacterial isolates F3, C1, C2, F6, and D6-W; bacteria were cultivated on PyOM agar and a rich medium (International Streptomyces Project 2, ISP2 agar) and assayed against *P. omphalodes* P1672; zones of inhibition from select *Paraburkholderia-*produced metabolites were larger from PyOM cultures compared to ISP2 cultures; isolate F6 cultivated on either medium did not inhibit *P. omphalodes*, while isolate D6-W inhibited *P. omphalodes* when cultivated on ISP2, but not on PyOM.

The pyrophilous fungus *P. omphalodes* has been observed to dominate postfire fungal communities, but then rapidly decline in abundance [7]. We hypothesized that specialized metabolite-mediated antagonism may be one factor contributing to *Pyronema*’s decline. Therefore, we screened a library of bacteria isolated from soil collected from Blodgett Forest Research Station, which underwent a prescribed burn in October 2018, for antifungal activity against *P. omphalodes* using an agar plug assay (Fig. 1A). To mimic environmental conditions, we grew the bacterial isolates on solid minimal medium (MM) containing PyOM as the sole carbon source. We also grew each strain in parallel on a rich medium (ISP2 agar), which contained glucose as the carbon source. For each isolate screened, we looked for differential antifungal activity from plugs that were taken from PyOM cultures compared to ISP2 cultures. We hypothesized that isolates exhibiting increased bioactivity when grown on PyOM were more likely to be sources of novel compounds, which may have remained undetected using traditional, rich medium-based laboratory screens.

Out of 38 bacterial isolates screened, 30 strains displayed antifungal activity against *P. omphalodes* (Table S3)*.* Five strains produced larger zones of inhibition when grown on PyOM compared to ISP2 (results for strains F3, C1, C2 shown in Fig. 1B), all of which were identified as members of the genus *Paraburkholderia* by sequencing their 16S rRNA genes. The results from this screen revealed that antifungal activity is prevalent among bacterial postfire soil isolates, and *Paraburkholderia* isolates are particularly noteworthy in their heightened activity during culturing on PyOM agar.

Given the observed antagonism of *Pyronema* by *Paraburkholderia*, we were curious about the abundance and growth dynamics of these two taxa in the environment. We re-analyzed 16S and Internal Transcribed Spacer (ITS) community sequencing datasets from two previously published time-series studies covering three distinct postfire sites, including the site from which the tested *Paraburkholderia* were isolated [11, 12]. These field sites represent different ecosystem types (mixed conifer forest and chaparral) and fire types (wildfire and prescribed fire). In all three sites, operational taxonomic units (OTUs) for the genus *Pyronema* and OTUs for the clade comprised of *Paraburkholderia*, *Burkholderia*, and *Caballeronia* (which cannot be delineated at the level of 16S gene sequence) were detected at high normalized relative abundance after fire (Fig. S1). Over the following ~1 year timeframe, abundances of both taxa were variable with a tendency to be anticorrelated.

### Discovery and structural elucidation of novel antibiotic surfactants: rhamnolipid methyl esters

**Figure 2:**
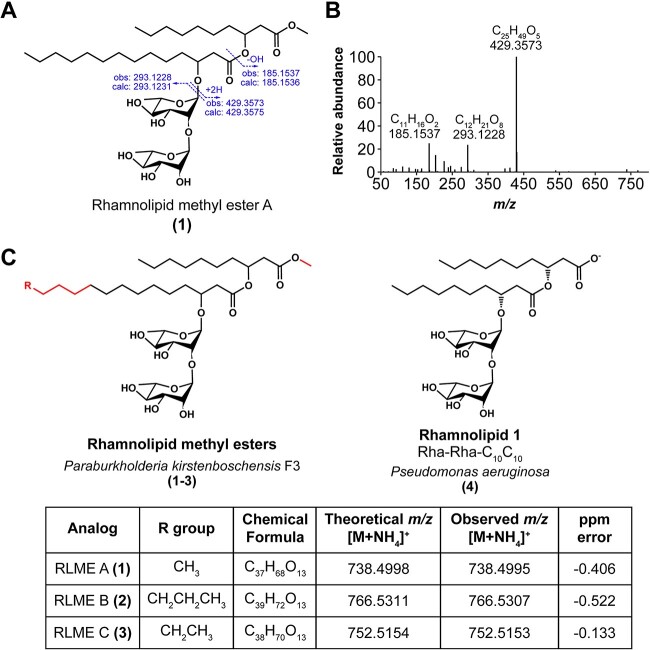
Structural elucidation of novel rhamnolipid methyl esters; (A) proposed chemical structure for the *P. kirstenboschensis* F3-produced RLME A; dashed arrows indicate theoretical molecular ion fragments that produce *m/z* values observed (obs) in the experimental data shown in (B) along with theoretically calculated values (calc); (B) HR-MS/MS fragmentation spectra obtained using collision energy of 15 eV; (C) rhamnolipid methyl esters produced by *P. kirstenboschensis* F3 with details for each analog, and the known Rhamnolipid 1 produced by *P. aeruginosa*; structural differences are highlighted in red; theoretical *m/z* values and ppm errors were calculated using the Barrow group online calculator tool; chemical formulas represent neutral species.

Based on the above antifungal screen, we selected isolate F3 as a promising candidate for further chemical analysis and investigation, with the aim to identify the molecule responsible for *P. omphalodes* inhibition. Isolate F3 is tentatively identified here as *Paraburkholderia kirstenboschensis* based on average nucleotide identity (ANI) with the reference genome (Fig. S2). We scaled-up cultivation of *P. kirstenboschensis* F3, extracted the spent medium with ethyl acetate, and subjected the crude extract to preliminary purification *via* solid-phase extraction. Subsequent antifungal assay-guided fractionation *via* semi preparative-scale reverse-phase high-pressure liquid chromatography led to the isolation of the primary active molecule (Fig. 2A). Positive-mode high-resolution mass spectrometry (HRMS) analysis revealed a peak for an ammonium adduct ion at *m/z* 738.4995, corresponding to a neutral species molecular formula of C_37_H_68_O_13_. The compound was proposed as a rhamnolipid through 1D and 2D nuclear magnetic resonance (NMR) spectroscopy and comparison with a previous reference (Table S4, Fig. S3–S6) [13]. The presence of the methyl ester was indicated by ^3^*J*-HMBC correlation from H-11 (*δ*_H_ 3.58) to C-1 (*δ*_C_ 170.4) (Fig. S6). The length of the two lipid chains, presence of rhamnose groups, stereochemistry, and connections between these moieties were further elucidated as detailed in the Materials and Methods (Fig. 2B, Table S4, Figs S3–S6). High-resolution tandem mass spectrometry (HR-MS/MS) analysis enabled the further identification of additional RLME analogs, with varying lengths in the second acyl chain (Fig. 2C, Fig. S7). These analogs were named RLME A-C according to decreasing observed relative abundances (Fig. S8). RLME B was also isolated and displayed comparable inhibitory bioactivity against *P. omphalodes* 1672 (Fig. S9). Together, these data indicated that the active molecules (1–3) were rhamnolipids carrying an unusual methyl ester modification where typical rhamnolipids, such as Rhamnolipid 1 produced by *Pseudomonas* spp. (4), terminate in a carboxyl group. This methylation, which alters the polarity of the molecule, is expected to impart altered surfactant properties to these RLMEs.

### Identification of a novel rhamnolipid methyltransferase encoded by *rhlM*

**Figure 3:**
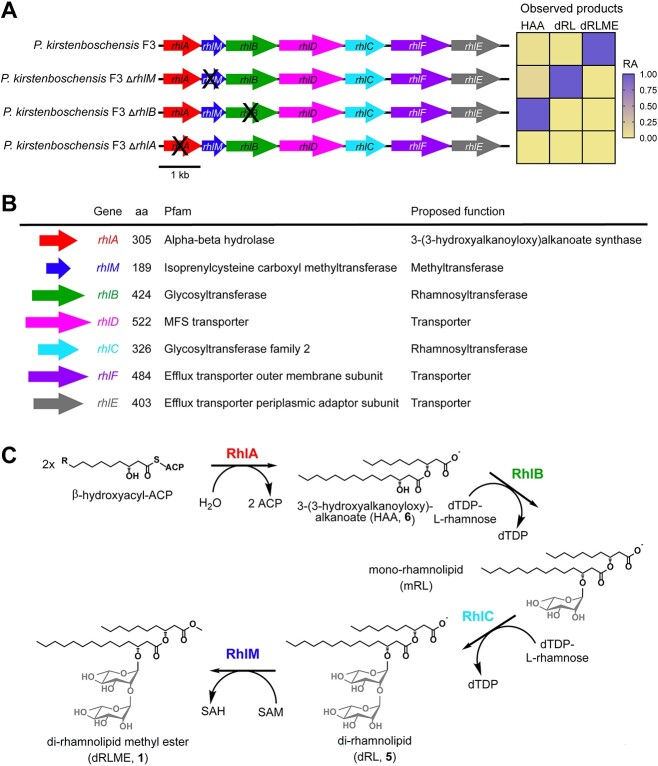
Rhamnolipid methyl ester biosynthesis genes are clustered in *P. kirstenboschensis* F3; (A) genotypes of WT and knockout mutants; heatmap of production of RLME and biosynthetic intermediates of *P. kirstenboschensis* F3 wild type and knockout mutants, observed using LC–MS; relative abundance (RA) of possible *rhl* pathway intermediates found in *P. kirstenboschensis* F3 knockout strains and wild type (WT), by extracted ion chromatogram peak area and normalized by RA within each strain (slate blue, more abundant; tan, less abundant); (B) genes in the RLME BGC with their amino acid (aa) length, Pfam annotation, and proposed function; the newly identified *rhlM* gene encodes for a putative Class VI integral membrane methyltransferase; (C) proposed pathway for biosynthesis of di-RLME A in *P. kirstenboschensis* F3; dRL, di-rhamnolipid; dRLME, dirhamnolipid methyl ester; ACP, acyl carrier protein; dTDP, deoxythymidine diphosphate; mRL, mono-rhamnolipid.

To identify the genes responsible for RLME biosynthesis, and in particular rhamnolipid carboxyl methylation, we sequenced the full genome of *P. kirstenboschensis* F3*.* A BLAST search of the *P. kirstenboschensis* F3 genome using *Pseudomonas aeruginosa rhl* gene homologs led us to identify the *rhl* gene cluster (Fig. 3A and B, Table S5). The uncharacterized gene downstream of *rhlA* was annotated as an isoprenylcysteine carboxyl methyltransferase (ICMT) family protein (locus_tag:RW095_02150). We hypothesized that the enzyme encoded by this gene, tentatively named *rhlM*, likely catalyzes the methylation of rhamnolipids to form rhamnolipid methyl esters.

To test the hypothesized role of *rhlM*, we used double allelic exchange to create a knockout mutant lacking the *rhlM* gene. Furthermore, to guide the full elucidation of the biosynthetic pathway, we created single mutants of *rhlA* and *rhlB* for chemical analysis of their intermediate products*.* We grew each mutant alongside wildtype *P. kirstenboschensis* F3 (WT), performed chemical extractions using ethyl acetate, and analyzed the extracts using liquid chromatography coupled with tandem mass spectrometry (LC–MS/MS).

Deletion of *rhlM* abolished the production of rhamnolipid methyl esters (Fig. 3A), while precursors including O-desmethyl rhamnolipid products and 3-(3-hydroxyalkanoyloxy)alkanoates (HAAs) were still produced (Fig. S10). Complementation of Δ*rhlM* with an *rhlM*-expressing plasmid rescued production of RLMEs, confirming the role of *rhlM* in methylation of rhamnolipid precursors. As predicted, in Δ*rhlB* strain extracts, HAAs were detected, while rhamnosylated products and methylated HAAs were not (Fig. 3A, Figs S10 and S11). No rhamnolipid pathway intermediates were detected in Δ*rhlA* extracts (Fig. 3A, Figs S10 and S11). These results confirmed the roles of *rhlA* and *rhlB* in 3-hydroxy-fatty acid esterification and rhamnosylation, respectively. These phenotypes were similarly rescued with their respective complementation on stable plasmids with constitutive expression (pBBR1-MCS5) (Fig. S10). Together, the observed intermediate products of Δ*rhlA*, Δ*rhlB*, and Δ*rhlM* allow us to propose rhamnolipid methylation by RhlM as the final step in the biosynthesis of RLMEs (Fig. 3C, Fig. S11).

Many methyltransferases, such as those of the ICMT family that includes RhlM, utilize S-adenosyl methionine (SAM) as a methyl donor. To further validate the biosynthetic origin of the carboxymethyl group of RLMEs, we performed a stable isotope labeling experiment using L-[D_3_]-methionine. Feeding L-[D_3_]-methionine to WT cultures led to incorporation of three deuterium atoms into RLMEs, as confirmed by the detection of a + 3 major isotopologue for each of the [M + NH_4_]^+^ and [M + Na]^+^ adducts via HR-MS/MS analysis of crude ethyl acetate extracts (Fig. S12). *P. kirstenboschensis* F3 also produces small quantities of O-desmethyl RLME (compound 5), and this mass feature did not show incorporation of any stable isotopes. Taken together, these results not only confirm that *rhlM* is responsible for rhamnolipid methylation, but further verify the activity of a SAM-dependent methyltransferase in RLME biosynthesis.

### *rhl* mutants display reduced antifungal activity

Having demonstrated the role of the genes *rhlA*, *rhlB*, and *rhlM* in RLME biosynthesis, we next sought to characterize the antifungal phenotype of each knockout mutant. Rhamnolipids (RLs) and HAAs, the respective major products that accumulate in Δ*rhlM* and Δ*rhlB* strains, are also known to exhibit antifungal activity [14–16]. To compare the antifungal activity produced by these mutant strains against strains producing RLMEs, we grew the WT and the mutants in parallel under consistent growth conditions and performed a plug assay.

**Figure 4:**
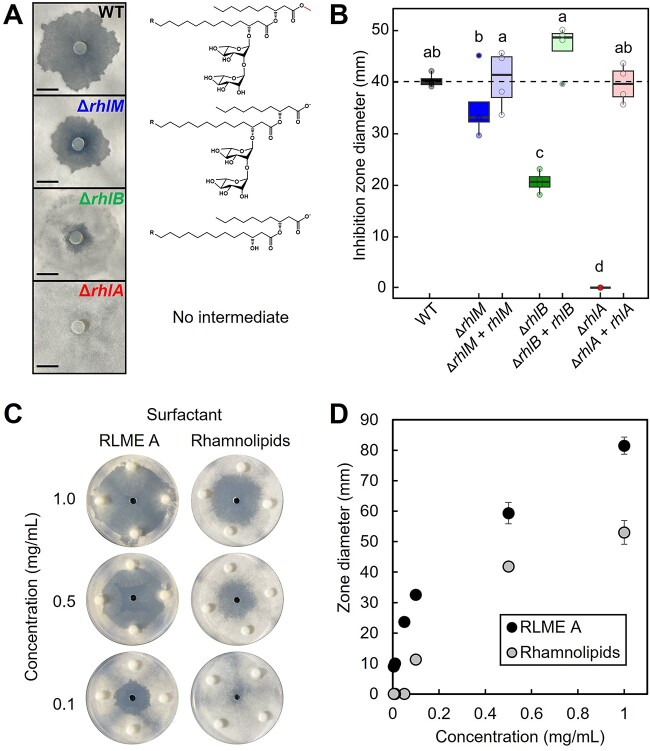
(A) *P. kirstenboschensis* F3 WT- or *rhl* mutant-conditioned plugs tested against *P. omphalodes*; images are representative of four biological replicates per strain and at least three independent experimental replicates; structures to the right represent the major *rhl* pathway product for each strain; the RLME methyl group is highlighted in red; scale bar is 1 cm; (B) clearance zone diameters for *P. kirstenboschensis F3* WT, *rhl* mutant strains, and genetic complement strains tested against *P. omphalodes*; data are representative of four replicates per strain; different letters indicate a statistically significant difference as determined by a one-way ANOVA and post hoc Tukey’s test (*P* < .05); dashed line indicates mean wildtype measurement; (C) inhibition of *P. omphalodes* using different concentrations of purified RLME A from *P. kirstenboschensis* F3 or Rhamnolipids (Di-Rhamnolipid dominant mixture) from *P. aeruginosa* (Sigma-Aldrich); compounds were solubilized and diluted in methanol and 15 μl of each solution was applied in the central well; images are representative of three technical replicates; (D) quantification of the inhibition zone diameters from tested RLME A and Rhamnolipids mixture; points represent the mean of three replicates for each condition, and error bars represent standard deviation.

As expected, the mutant Δ*rhlA* displayed no antifungal activity, while knocking out the intermediate genes *rhlM* and *rhlB* reduced antifungal activity but did not completely abolish it (Fig. 4A). Plugs taken from the Δ*rhlM* strain displayed a 26.0% reduction in average zone diameter, and plugs from Δ*rhlB* displayed a 95.7% reduction in average zone diameter. Complementation of the *rhlM*, *rhlB* or *rhlA* gene in the corresponding deletion mutant restored antifungal activity to WT levels, demonstrating that the observed phenotype is due to RLME production (Fig. 4B). These data indicate that the inhibitory activity of *P. kirstenboschensis* F3 originates solely from products of the *rhl* pathway. Comparison between wildtype (WT) and Δ*rhlM* strains suggests that carboxyl methylation significantly enhances antifungal activity of rhamnolipids.

### Rhamnolipid methyl esters exhibit stronger antimicrobial activity than rhamnolipids from *P. aeruginosa*

Although the agar plug assays above suggest that RLMEs more strongly inhibit *P. omphalodes* than unmethylated rhamnolipids, live cell cultures may exhibit variability in both metabolite concentrations and compositions. This variability complicates the analysis of structural differences and associated antifungal activity in WT and mutant strains. To circumvent this challenge, we assayed purified RLME A and a commercial standard of rhamnolipids (di-rhamnolipid dominant mixture) from *P. aeruginosa* for antifungal activity against *P. omphalodes*, at concentrations ranging from 0.005 mg/ml to 0.1 mg/ml.

In all cases, RLME A was active at lower concentrations than the rhamnolipid mixture to create a zone of inhibition against *P. omphalodes*. Zones of inhibition are observed for RLME A at concentrations as low as 0.005 mg/ml, while a 20-fold higher concentration of 0.1 mg/ml was required for rhamnolipids to produce a zone of inhibition (Fig. 4C). At equal concentrations, the zones of inhibition observed for RLME A were 41.8%–186.8% larger than those observed for rhamnolipids (Fig. 4D). These findings indicate that RLME A has more potent activity against *P. omphalodes* than rhamnolipids from *P. aeruginosa*.

Given that our initial antifungal screen included only one fungal isolate (*P. omphalodes* 1672) as an indicator strain, we assayed other *Pyronema* isolates as well as postfire fungal isolates of other genera for inhibition by *P. kirstenboschensis* F3, using cultures of F3 Δ*rhlA* as a negative control. RLMEs inhibited seven out of eight *Pyronema* sp. isolates from other postwildfire sites in California and Oregon; however, other postfire fungi were not affected (Table S6). We further assayed *P. kirstenboschensis* F3 for inhibition of a set of postfire bacteria representing diverse phyla and families. Among the isolates tested, only *Amycolatopsis* spp. were affected (Table S7). When purified RLME A and RLME B were applied to solid *Amycolatopsis* cultures, the RLMEs appeared to create a zone of clearance and suppress aerial hyphae development (Fig. S13). Similar to *P. omphalodes*, *Amycolatopsis* spp. were more susceptible to RLMEs than the standard rhamnolipids when tested at equal concentrations (Fig. S13).

### RLME and intermediates promote *P. kirstenboschensis* F3 motility *via* formation of a surfactant front

Several studies have linked rhamnolipid and HAA production with bacterial swarming motility in *P. aeruginosa* and *Burkholderia* species [17–19]. Specifically, evidence suggests that these molecules act as surfactants to promote swarm expansion [20]. We sought to explore whether RLMEs and their precursors were connected to swarming motility of *P. kirstenboschensis* F3. We tested the impact of RLMEs on *P. kirstenboschensis* F3 motility in the WT and in knockout mutants lacking *rhlM*, *rhlB*, and *rhlA*. We tested the motility phenotypes of these strains on a rich ISP2 medium containing 0.25% agar and on a MM containing 0.5% agar. After incubation, we visualized the surfactant zones on MM + 0.5% agar plates using atomized oil spray [21].

**Figure 5:**
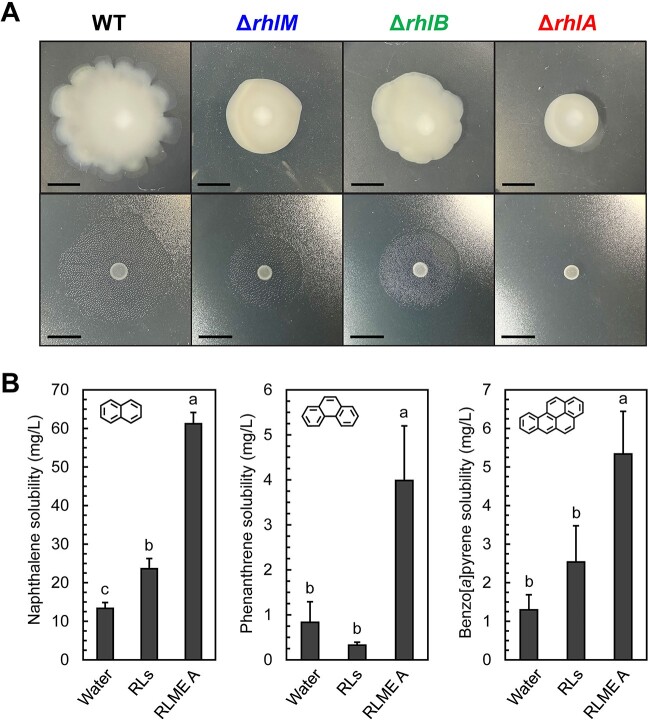
(A) Motility phenotypes (top) and surfactant zone production (bottom) for *P. kirstenboschensis F3* WT and *rhl* mutants; images are representative of at least five biological replicates per strain and at least three independent experimental replicates; scale bar is 1 cm; (B) solubilization of naphthalene (left), phenanthrene (center), and benzo[*a*]pyrene (right) in water supplemented with either 500 mg/l RLME A (1) from *P. kirstenboschensis* F3 or 500 mg/l Rhamnolipids (RLs, Di-Rhamnolipid dominant mixture, Sigma-Aldrich) from *P. aeruginosa*; different letters indicate a statistically significant difference as determined by a one-way ANOVA and post hoc Tukey’s test (*P* < .05).

We observed that the WT strain was motile and spread out radially on the agar surface with a pronounced undulate margin (Fig. 5A). Both Δ*rhlM* and Δ*rhlB* strains exhibited reduced motility, with 51.7% and 30.6% reductions in average swarming diameter, respectively. Furthermore, the Δ*rhlM* strain swarm edges were entire rather than undulate, while the Δ*rhlB* strain swarm edges were only slightly undulate. The atomized oil assay revealed that, like WT, Δ*rhlM* and Δ*rhlB* strains produced a surfactant front, but they were reduced in diameter by 9.4% and 9.0%, respectively (Fig. 5A). Deletion of *rhlA* completely abolished swarming motility, and no surfactant front was observed when Δ*rhlA* MM plates were sprayed with atomized oil. Reductions in swarming for each mutant were ameliorated with genetic complementation (Fig. S14). These findings indicate that RLMEs and their precursors are directly involved in *P. kirstenboschensis* F3 swarming motility on agar surfaces *via* the formation of a surfactant front.

### Rhamnolipid methyl esters improve aqueous solubilization of polycyclic aromatic hydrocarbon compounds

*P. kirstenboschensis* F3 was isolated from a burned soil environment, in which a substantial amount of carbon is present in the form of pyrolyzed organic matter (PyOM, or char). PyOM consists of a complex mixture of PAHs, which are hydrophobic substrates and thus poorly bioaccessible [22]. Analysis of the *P. kirstenboschensis* F3 genome showed that it possesses full or partial pathways for degradation of benzoate, catechols, and other common intermediates of aerobic catabolism of aromatic substrates (Figs S15 and S16). Previous studies have shown rhamnolipid-enhanced solubilization of hydrocarbons, such as alkanes [23, 24] and PAHs [25–27]. Synthetic rhamnolipid methyl esters were also shown to further enhance hydrocarbon solubilization [23]. Thus, we sought to investigate the ability of rhamnolipid methyl ester A (RLME A) to solubilize PAHs and assess its performance alongside a commercial rhamnolipids standard.

We tested purified RLME A for solubilization of three PAHs (naphthalene, phenanthrene, and benzo[*a*]pyrene) in comparison to the rhamnolipids. Biosurfactant was supplied to the mixtures at a concentration exceeding literature values of critical micelle concentrations (CMCs) for rhamnolipids, taking into account lower predicted CMC values for RLMEs [23]. RLME A improved aqueous solubilization of each individual PAH by nearly 5-fold compared to base solubilities in water (Fig. 5B, S17), while rhamnolipids improved solubilization by only ~2-fold. These data indicate that RLMEs significantly enhance the solubility of these PAHs to a greater extent than typical rhamnolipids.

### Rhamnolipid methyl esters enable bacterial motility and inhibit *P. omphalodes* invasion in a spatially defined pyrolyzed organic matter substrate environment

**Figure 6:**
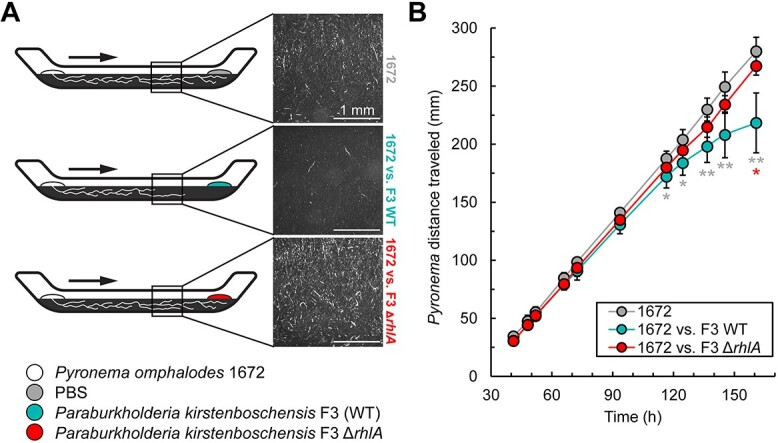
Race tube assay for inhibition of *P. omphalodes* 1672 on pyrolyzed organic matter (PyOM); (A) schematic for race tube assay; *P. omphalodes* was inoculated at one end of the race tube opposite from either PBS (negative control), *P. kirstenboschensis* F3 (F3) wildtype (WT), or F3 Δ*rhlA*; arrows indicate direction of *P. omphalodes* growth; images were acquired 16.5 cm from the *P. omphalodes* inoculation point and taken from above; scale bar is 1 mm; (B) *P. omphalodes* growth over time, measured by distance from point of inoculation; data points are mean values of either six biological replicates (1672 vs. F3 WT and F3 Δ*rhlA*), or five biological replicates (1672 alone); error bars represent standard deviation; statistical significance was measured by Welch’s *t*-test between two conditions at a single timepoint; gray asterisks indicate statistically significant differences between 1672 alone and when inoculated across from F3 WT; red asterisks indicate statistically significant differences between 1672 inoculated across F3 WT and when inoculated across F3 Δ*rhlA*; ^*^P ≤ .05*,*  ^*^^*^P ≤ .01.

The RLMEs produced by *P. kirstenboschensis* F3 exhibit multiple types of activity, including inhibition, motility promotion, and PAH solubilization. We next sought to assess the potential ecological relevance of these functions within a simple environment designed to incorporate postfire substrates and microbial patchiness. To do so, we used race tubes, glass cylinders typically used to monitor the unidirectional growth of filamentous fungi, containing a uniform layer of agar with PyOM as the sole carbon source (Fig. 6A). *P. omphalodes* 1672 and *P. kirstenboschensis* F3 were then inoculated at opposite ends of each race tube and allowed to grow toward one another. To assess the potential role of RLMEs, we also inoculated race tubes with the F3 Δ*rhlA* strain in place of WT *P. kirstenboschensis* F3. A set of tubes with phosphate-buffered saline (PBS) included in lieu of bacteria were also used as negative controls.

Within the race tube environment, both *P. omphalodes* and *P. kirstenboschensis* F3 were able to grow using PyOM as the sole carbon source. When placed opposite from F3 WT, *P. omphalodes* growth was linear and consistent until around 117 h postinoculation (Fig. 6B). By 160 h postinoculation, *P. omphalodes* growth in these tubes was significantly curtailed, and the mycelium density was also heavily reduced (Fig. 6A). In contrast, *P. omphalodes* grew unabated when it was inoculated alone or opposite from the F3 Δ*rhlA* strain. Furthermore, F3 WT displayed a biosurfactant-promoted motility phenotype that was not observed in the Δ*rhlA* mutant (Fig. S18). Altogether, these findings demonstrate that F3-produced biosurfactants were able to significantly inhibit fungal invasion and enable bacterial motility in a PyOM-dominated nutrient environment.

### RhlM is a putative S-adenosyl methionine-utilizing integral membrane carboxyl methyltransferase

Multiple lines of investigation in this study point toward the carboxymethyl group in RLMEs as a key functional contributor to their surface activities, with relevance for antifungal activity, motility, and solubilization of potential PAH nutrient substrates. The *P. kirstenboschensis* F3 RhlM enzyme belongs to the ICMT family of integral membrane methyltransferases, which are predicted to accommodate both an amphiphilic substrate and the polar cofactor SAM. Aside from archetypal ICMTs, which are known to methylate the carboxyl group of prenylated protein substrates in eukaryotes, no other methyl acceptor substrates have been identified for proteins of this family. As a result, understanding the function of RhlM is of high interest. Thus, we sought to further explore RhlM with respect to its structure, substrate binding, and evolutionary context *in silico*.

**Figure 7:**
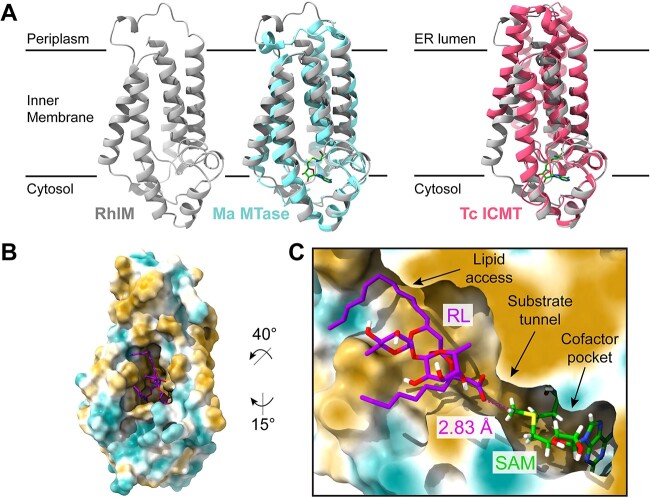
(A) AlphaFold model for RhlM (gray) alone, aligned with *M. acetivorans* methyltransferase (PDB: 4a2n, cyan), and aligned with *T. castaneum* ICMT (PDB: 5vg9, pink); SAH is shown in green; the membrane is indicated by two solid horizontal lines; (B) surface representation of the Rank 1 pose from Maestro ligand docking in RhlM, using an applied positional constraint for substrate carboxylate O and SAM methyl C; docked pose was verified by molecular dynamics using Desmond; surface is colored by molecular lipophilicity potential (gold = lipophilic, cyan = hydrophilic); (C) cross-section of RhlM with substrate and cofactor bound; the upper hydrophobic region provides access for the lipid region of the RL substrate, while the lower region accommodates the carboxylate terminus, optimally positioning the carboxylate for methyl transfer from the SAM cofactor; measured C-O distance is shown in magenta.

To structurally characterize RhlM *in silico*, we generated a protein model for RhlM using AlphaFold (Fig. 7A, Fig. S19), which we aligned with the two crystallized members of the ICMT protein family, the archaeal Ma MTase (PDB: 4a2n) and eukaryotic *Tribolium castaneum* ICMT (PDB: 5vg9) [28, 29]. The model of RhlM aligned well with both reported crystal structures, with pruned RMSD values of 1.118 Å and 0.793 Å, respectively (Fig. 7A, Table S8). After docking the S-adenosyl homocysteine (SAH) cofactor into our RhlM model, we observed that SAH bound in similar conformations in RhlM and Ma MTase, as did SAM. Two residues (E162 and H122) form interactions with SAM, closely resembling ligand interactions observed in SAH-bound Ma MTase and SAH-bound Tc ICMT (Fig. S20A and B) [28, 29]. Clustal Omega sequence alignments of RhlM with different ICMTs and other orthologs also demonstrated that these are conserved residues in the putative SAM cofactor binding pocket (Fig. S20C).

Chemical analyses of extracts from *rhl* pathway knockout mutants (Fig. 3A, Figs S10 and S11) indicate that the major methyl accepting substrate of RhlM is likely the di-rhamnolipid Rha-Rha-C_14_-C_10_ (RL, compound 5). To investigate binding of RL within RhlM, we performed sequential ligand docking experiments in Glide first with SAM followed by RL. We next performed a molecular dynamics simulation in Desmond, using RhlM modeled within a lipid membrane environment, to refine docked poses of RL within RhlM. These simulations place the RL lipid chains in contact with hydrophobic residues that form a lipid-binding region (Fig. 7B and C, Fig. S21). This lipid-binding pocket appears to be large and flexible enough to accommodate substrates with variable lipid chain lengths, in line with the observation of different RLME analogs produced by *P. kirstenboschensis* F3. This arrangement of compound 5 positions the RL carboxylate O within the active site in proximity to the SAM methyl C (~3 Å) for methyl transfer [30]. Taken together, these *in silico* results suggest a plausible mechanism for methylation of di-rhamnolipids by RhlM and set the stage for further structural and biochemical characterization of this novel enzyme.

### Prevalence and evolution of rhamnolipid methyl ester biosynthesis across *Burkholderia*  * sensu lato*

**Figure 8:**
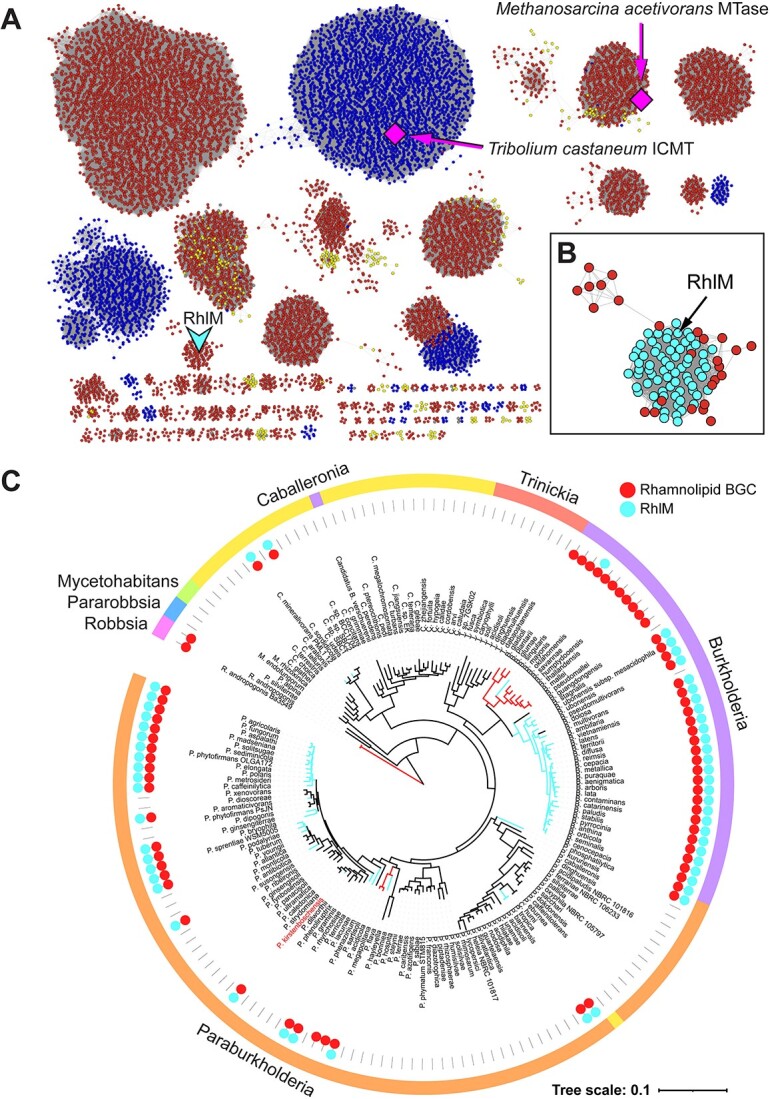
(A) Sequence similarity network (SSN) for ICMT protein family; nodes are colored by domain: red, Bacteria; blue, Eukarya; yellow, Archaea; gray, unclassified; cyan V-shape indicates *P. kirstenboschensis F3* RhlM, magenta diamonds indicate that a crystal structure is available; clusters containing three or fewer nodes are omitted for clarity; (B) enlarged view of Cluster 14 from the SSN; cyan-colored nodes indicate that the RhlM homolog sequence is found within a rhamnolipid biosynthetic context, and that the strain is of the family *Burkholderiaceae*; (C) species tree of the BSL clade, showing presence of the rhamnolipid BGC (red circle) and RhlM (cyan circle) within genomes of each species identified using BLAST; the outgroup (*Bacillus subtilis* str. 168) was manually removed to facilitate visualization.

To better define RhlM within the context of the ICMT protein family, we used the Enzyme Function Initiative’s Enzyme Similarity Tool (EFI-EST) [31] to generate a sequence similarity network (SSN) using all 14 240 sequences with the ICMT Pfam ID PF04140, with the RhlM sequence manually included (Fig. 8A). This network analysis revealed that the *P. kirstenboschensis* F3 RhlM sequence was part of a distinct cluster (Cluster 14) composed of RhlM homologs predominantly found in the context of rhamnolipid biosynthesis in *Burkholderia*, *Paraburkholderia*, and *Caballeronia* (Fig. 8B, Fig. S22). Phylogenetic analysis of Cluster 14 members indicates that proteins closely homologous to RhlM are also found in distantly related bacterial taxa, such as *Flavobacteriia* and *Chitinophagia*. RhlM homologs are also found in more closely related *Rhodanobacter* spp., where the *rhlM*-like gene is located near a fatty acid hydroxylase and an *rhlA*-like alpha–beta hydrolase (Fig. S21).

Broadly, the full SSN comprises 230 clusters and is dominated by clusters of bacterial origin, with a few eukaryotic bacterial/archaeal subnetworks also present. This SSN analysis illustrates that ICMT paralogs are widespread in bacteria. These findings hint at a wide potential for future study within this enzyme class, with RhlM providing a starting point for elucidating the function of these enzymes across the bacterial domain.

In addition to our SSN analysis, a recent bioinformatic survey also identified rhamnolipid biosynthetic gene clusters (BGCs) containing RhlM-like sequences across *Burkholderiaceae* genomes [32]. These findings raise the possibility that RLME biosynthesis may be common among *Paraburkholderia* and the broader *Burkholderia* sensu lato (BSL) clade. To investigate the prevalence of *rhlM*-dependent RLME production in the *Paraburkholderia*, we examined six additional *Paraburkholderia* isolates from burned soil that also inhibited *P. omphalodes*. Compound 1 was detected by LC-HRMS and MS/MS in extracts of all six isolates, and PCR amplification of genomic DNA from all six strains revealed the presence of the associated *rhlM* gene (Table S9).

To further examine the prevalence of RLME biosynthesis across the BSL clade, we performed a bioinformatic analysis of all species from the seven genera of BSL using genome sequences available in the NCBI database. We constructed a species tree using one representative genome for each species and mapped the presence or absence of the rhamnolipid BGC and RhlM within each genome (Fig. 8B). Rhamnolipid BGCs were found in four of the seven BSL genera. The most ancient clade, containing *Robbsia* spp., possesses genes for rhamnolipid biosynthesis but lacks RhlM. Rhamnolipid biosynthesis is rare in *Caballeronia* spp. but highly prevalent across the genus *Burkholderia*, with 95% of *Burkholderia* spp. possessing *rhl* genes. The *Burkholderia* genus is divided into two distinct subclades, one of which contains strains capable of RLME biosynthesis, while strains in the other subclade lack RhlM with one exception. In contrast, the distribution of *rhl* biosynthesis genes is less consistent across *Paraburkholderia* spp. However, of those that possess the *rhl* BGC, the majority (92%) also have RhlM. Altogether, these results illustrate a high potential for RLME production across the BSL clade and point toward a complex evolutionary history of RL/RLME biosynthesis.

## Discussion

The postfire soil environment is a challenging place for microorganisms to live and thrive—and one in which competition for limited resources is likely fierce as the microbial community successively assembles. Aside from biotic competition, postfire microorganisms face numerous other challenges, including increased hydrophobicity and poor nutrient solubility [33–36]. However, our understanding of the mechanisms by which postfire microorganisms adapt to their perturbed environments remains limited.

Here, we report a set of rhamnolipid biosurfactants with an uncommon carboxymethylation, the rhamnolipid methyl esters (RLMEs), produced by a postfire bacterium, *P. kirstenboschensis* F3. These RLMEs inhibited the growth of a common pyrophilous fungus in a spatially defined PyOM substrate environment, promoted bacterial motility, and enhanced PAH solubility. In addition, we identified the genes required for RLME biosynthesis and found that a novel methyltransferase, termed RhlM, is required for the installation of the unusual carboxymethyl group found in these molecules. RhlM is the first characterized bacterial representative of a class of integral membrane methyltransferases that is widespread in bacteria. In sum, this study provides an example of specialized metabolism that may confer multiple adaptive advantages to postfire bacteria. Furthermore, this work highlights the potential in exploring perturbed environments for novel natural products and their associated enzymology.

### Probing perturbed environments leads to antifungal discovery

We employed an ecologically informed screening strategy intended to bias our findings toward unusual compounds. This approach began with isolates sourced from a postfire environment, which we cultivated using a medium containing an environmentally relevant carbon source in the form of pyrolyzed organic matter (PyOM). We screened for antifungal activity using an indicator strain, *P. omphalodes*, which is found in high abundance in postfire soils [7, 37]. We specifically looked for antifungal activity that was enhanced when the producing isolates were grown on PyOM, as opposed to a rich medium. Thus, at each step, we designed this strategy to increase the probability of identifying molecules that might carry ecological significance. This bioprospecting strategy led to the identification of the RLMEs. This approach represents a model framework to guide future discovery of bioactive compounds from perturbed environments, including molecules like RLMEs that possess modified chemical properties and/or function under unusual environmental conditions.

### Biosynthesis of rhamnolipid methyl esters involves an integral membrane methyltransferase

Rhamnolipid methyl esters are members of a known class of rhamnolipid biosurfactants, which are thought to fulfill several different roles for their producing organisms. Rhamnolipid 1, first discovered from *P. aeruginosa* [38], is known to exhibit antimicrobial properties and promote bacterial swarming motility [14–19]. Rhamnolipids have also been identified as potential mediators for uptake of hydrophobic substrates by *P. aeruginosa* and consortia [23, 39–41]. As a result of carboxyl methylation, the typically anionic lipid region of rhamnolipids is neutralized in RLMEs, rendering this region even more lipophilic. This key difference likely imparts stronger surface-active properties upon RLMEs, which may play a role in the enhanced antifungal activity, swarming motility, and PAH solubilization demonstrated here for RLMEs from *P. kirstenboschensis* F3.

The enhanced surface activity of rhamnolipid esters has been previously established through studies that utilized synthetic RLMEs [23]. However, to our knowledge, naturally occurring rhamnolipid methyl esters had been reported only once prior to posting of this article as a preprint [42], without further verification of the biological origin of the appended methyl group. The identification of a natural producer of RLMEs, as well as elucidation of RLME biosynthesis, is therefore of notable interest for advancing the field of microbial biosurfactants as well as for industrial applications. We note that although an abundance of reports has focused on altering production titers [43–45], and structural components of rhamnolipids [46–48], none have provided a biological means toward esterification of the carboxy terminus. This work lays the foundation for the development of heterologous expression or RhlM-dependent biocatalytic approaches for optimized production of RLMEs with altered chemical properties.

The rhamnolipid methyltransferase identified here, RhlM, is a member of a larger group of proteins known as the ICMT family. This family includes only two crystallized representatives; a eukaryotic ICMT [29] and an archaeal ortholog from *Methanosarcina acetivorans* [28], whose methyl-accepting substrate has not been determined. Thus, although some eukaryotic and archeal ICMT family proteins have been investigated, the functions of these proteins in bacteria remained unexplored. The designation of RhlM as a member of the ICMT protein family led us to further investigate the prevalence of these enzymes in bacteria. Our sequence similarity analysis of over 14 000 proteins revealed a large number of ICMT paralogs that form many discrete clusters across the bacterial domain.

The RhlM sequence was grouped as a part of a well-resolved cluster (Cluster 14). Genome neighborhood analysis of Cluster 14 sequences revealed that the majority of these genes all lie within rhamnolipid biosynthetic operons in the genomes of *Paraburkholderia*, *Burkholderia*, and *Caballeronia*, suggesting that RLME production is common within these clades. Bioinformatic analysis of the broader BSL clade confirms this observation. These findings expand on a recent bioinformatic survey that identified many *Burkholderiaceae* genomes harboring RhlM-like sequences [32]. In further support of this notion, Gauthier *et al*. recently reported a third example of a RMLE produced by a species of *Burkholderia* [49]. Taken together, these results and phylogenetic analyses suggest that rhamnolipid biosynthesis is ancient within BSL, and that across the known species diversity within the BSL clade, RLME production is more common than the production of typical rhamnolipids.

Among all known methyltransferases, ICMT family proteins are uniquely transmembrane in nature, while all others are soluble enzymes [50]. Our modeling studies with RhlM highlight the features of these enzymes that are relevant to their function in methylating amphiphilic substrates. For example, the lipid moieties of rhamnolipid intermediates synthesized in the cytosol are likely to partition to the inner membrane, where they would have access to the lipid-binding tunnel leading toward the RhlM active site. This arrangement may facilitate the methylation of a wide array of amphiphilic substrates by other bacterial ICMTs, reflected by the manifold clusters of ICMT family proteins present in our sequence similarity analysis. In this regard, RhlM serves as a pivotal inroad into understanding the likely diverse functions of ICMT family enzymes in bacteria.

### Ecological implications for biosurfactant production within postfire soil communities

The array of challenges presented by postfire environments likely drives multifunctional adaptations in pyrophilous microorganisms. The results presented here support a model in which RLMEs are calibrated to function in postfire environments as mediators of interference competition, enhancers of motility, and potentiators of nutrient solubility.

The pyrophilous fungus *P. omphalodes* is a dynamic member of the postfire microbial community, sometimes achieving relative abundances reaching 60.34% after fire before subsequently declining [7]. This decline may be influenced by a host of factors, including interference competition mediated by chemical antagonism from an array of competitors. This hypothesis motivated our choice to use *P. omphalodes* as an indicator organism in our screens to identify antimicrobials made by other postfire organisms. Using this framework, we arrived at RLME biosurfactants as noteworthy candidates for *Pyronema* inhibition *in situ*. The demonstration of RLME-dependent growth inhibition of *Pyronema* in our race tube experiments (Fig. 6) highlights the possibility that *Paraburkholderia* may use RLMEs to limit encroachment of competitors in patchy, PyOM-enriched soil environments. We also noted that in three amplicon sequencing datasets across diverse ecosystems and fire types, relative abundances of *Pyronema* and the clade comprised of *Paraburkholderia*, *Burkholderia*, and *Caballeronia* tended to increase after fire and then appeared to be anticorrelated in the following year. Although we would caution against making strong conclusions based on this limited number of community studies, these results are consistent with the notion that *Paraburkholderia* may antagonize *Pyronema* in postfire environments. Finally, beyond the original *P. omphalodes* 1672 indicator strain, seven *Pyronema* isolates from five postfire sites, as well as *Amycolatopsis* spp. from two postfire sites, were also inhibited by RLMEs made by *P. kirstenboschensis* F3. These results are consistent with the idea that RLMEs may have a broad function in interference competition among postfire community members.

Rhamnolipids are reported to display a wide array of antibiotic activity, which include anti-oomycete activity against zoospores *via* lysis and antifungal activity of plant pathogens *via* growth inhibition [14, 51]. We found that RLMEs produced larger zones of inhibition against both *P. omphalodes* and *Amycolatopsis* spp. compared to typical rhamnolipids (Fig. 4, Fig. S13). The methyl ester group and R chain length likely alter the physicochemical properties (e.g. solubility) of RLMEs compared to the mixture of RLs from *P. aeruginosa.* Whether this enhanced activity results from superior diffusibility on hydrophobic surfaces or other mechanisms remains a subject for further investigation.

Beyond competition, microorganisms face increased hydrophobicity owing to PyOM in burned soils, which likely limits dispersal to, and colonization of, new microniches [36]. Here, we demonstrated that motility of *P. kirstenboschensis* F3 on agar surfaces, including PyOM agar, was dependent on products of the *rhl* biosynthetic pathway, which include HAAs, RLs, and RLMEs. This finding is broadly in line with previous studies showing that rhamnolipids enable swarming motility of *Pseudomonas* and *Burkholderia* species on similar surfaces [17, 52, 53]. Although HAAs and RLs enabled bacterial motility, only RLME production promoted maximal motility for *P. kirstenboschensis* F3. We hypothesize that production of enhanced biosurfactants like RLMEs may have strong implications for bacterial motility and colonization within hydrophobic burned soils. However, to our knowledge, swarming motility mediated by rhamnolipids has not been investigated in terrestrial environments. Efforts aimed at quantifying the advantages conferred by biosurfactants on bacterial motility in burned soils are therefore of keen interest going forward.

Postfire microorganisms must also contend with the additional challenge of nutrient limitation in burned soils, as a major component of the carbon is present in the form of PAHs associated with PyOM. These aromatic compounds are not only difficult to degrade, but their hydrophobicity decreases their bioavailability. Previous studies have shown that rhamnolipids can enhance the solubilization and degradation of PAHs [40, 54, 55]. We hypothesized that RLMEs would likely exhibit increased ability to solubilize PAHs compared to typical rhamnolipids due to their methyl ester functionality, which neutralizes the anionic carboxylate group and would thereby increase the polarity differential between the surfactant’s hydrophilic and lipophilic regions. Our results indicate that indeed RLMEs were significantly better at solubilizing three PAHs of varying complexity. Although we observed greater solubility enhancement using RLMEs compared to rhamnolipids, these differences may be attributable to increased surface activity from carboxyl methylation as well as the ≥4-carbon longer acyl chain present in RLMEs from *P. kirstenboschensis* F3. Dissecting the relative contributions of these chemical modifications will require further investigation. In addition, the *P. kirstenboschensis* F3 genome features, several dioxygenase genes likely involved in the oxidative catabolism of aromatic molecules (Figs S15 and S16). Collectively, these results are consistent with a robust role for RLMEs in the local solubilization of PAHs, which may serve as growth substrates for *P. kirstenboschensis* F3.

The multifunctional nature of RLMEs revealed by this work aligns with a growing body of evidence showing that many molecules initially regarded as antimicrobials may in fact play diverse roles for their producers, and may have variable impacts across microbial communities [8, 10, 56]. A key recent example includes phenazines produced by *Pseudomonas* species that influence iron and phosphate bioavailability, but also exhibit antimicrobial activity [9]. The possibility that RLMEs may serve as public goods for some community members by enhancing motility or nutrient access, while they may be detrimental to others (e.g. *Pyronema, via* antifungal activity), warrants further exploration in the context of postfire community assembly and succession.

## Materials and methods

### Strains and growth conditions

Bacterial strains and oligonucleotides used are listed in Tables S1 and S2. Bacterial strains were cultured in ISP2 broth (malt extract 10 g/l, yeast extract 4 g/l, dextrose 4 g/l) or on ISP2 agar (ISP2 broth plus agar 18 g/l) unless otherwise indicated. Fungal strains were cultured on Vogel’s Minimal Medium (VMM) agar [57] or CMYM agar (20 g/l corn meal agar, 1 g/l yeast extract, 1 g/l malt extract). *Paraburkholderia* spp. and other postfire bacteria were initially isolated from burned soil samples collected from Blodgett Forest, using an enrichment method on PyOM agar (1.5 mM potassium phosphate monobasic, 4.7 mM ammonium chloride, 6.7 mM potassium chloride, 1 mM calcium chloride, 17 mM sodium chloride, 3 mM magnesium chloride, 20.6 mM sodium sulfate, 7.5 g/l noble agar, 0.5 g/l ground PyOM [Eastern White Pine wood pyrolyzed at 350°C] [5], 7.5 μM iron(II) chloride, 0.8 μM cobalt(II) chloride, 0.5 μM manganese(II) chloride, 0.5 μM zinc(II) chloride, 0.1 μM boric acid anhydrous, 0.15 μM sodium molybdate dihydrate, 0.1 μM nickel(II) chloride, 11 nM copper(II) chloride, 58 nM 4-aminobenzoic acid, 8 nM D(+)-biotin, 164 nM nicotinamide, 42 nM D(+)-pantothenic acid hemicalcium, 83 nM pyridoxamine dihydrochloride, 59 nM thiamine hydrochloride, 37 nM cobalamin [Vitamin B12]). Initial screening of burned soil isolates was performed using ISP2 agar and PyOM agar.

### Antifungal screen for inhibition of *P. omphalodes*

Bacterial strains assayed for inhibition of *P. omphalodes* are listed in Table S3. Strains were selected to represent a range of phylogenetic diversity as well as source site, depth, and collection date. Bacterial isolates were cultured in liquid media for 2 days at 30°C. Cells were pelleted, washed twice in PBS, resuspended in 500 μl 0.1% PBS, and 500 μl was spread on ISP2 agar or PyOM agar (60 × 15 mm) using sterile beads, in triplicate. Plates were incubated for 5 days at 30°C. *P. omphalodes* was grown on VMM agar for 3 days at room temperature (RT) in standard 100 × 15 mm Petri plates. An agar plug from a plate of *P. omphalodes* was placed at the center of a fresh VMM agar plate, and agar plugs from each plate of bacteria were placed 2 cm from *P. omphalodes*. The presence (a zone of clearing) or absence (fungal growth around the agar plug) of antifungal activity was recorded after 3 days at RT. Measurements of zones of clearance were recorded. Agar plugs from uninoculated agar plates were used as negative controls. Chemical extracts and fractions were resuspended in methanol to an approximate concentration of 1 mg/ml. Purified compounds and standards were resuspended in methanol and diluted to the appropriate concentration. For all solutions, 15 μl was spotted in a 1cm-wide circular well.

### Plug assay screen for inhibition of postfire fungal and bacterial isolates

Cultures of *P. kirstenboschensis* F3 wildtype and Δ*rhlA* were prepared as described above. Fungal indicator strains were cultured at RT on either VMM agar (ascomycetes) or CMYM agar (basidiomycetes) in standard 100 × 15 mm Petri plates at RT until mycelia covered the whole plate, at which point four 0.5 cm-wide plugs were removed and placed on a fresh VMM or CMYM plate and evenly placed 3 cm from a central *Paraburkholderia* plug. Plug assay plates were incubated at RT until fungal growth covered the entire plate or a zone of inhibition was clearly observed.

Bacterial indicator strains were cultured in liquid media for 16–20 h at 30°C. About 50 μL of culture was spread across an ISP2 plate (60 × 15 mm) using sterile glass beads. Agar plugs from *Paraburkholderia* cultures were placed at the center of each plate. Pure compounds were assayed as described above.

### 16S/ITS amplicon community sequencing analysis

ITS and 16S raw sequence reads were downloaded from published NCBI SRA Accessions PRJNA761539 [12], and PRJNA835883 and PRJNA835896 [11]. *P. kirstenboschensis* F3 was isolated from postfire soil sample collected from the low-intensity prescribed fire site. These data were processed following the pipeline previously described in Fischer *et al*. . Briefly, 16S reads were processed using the QIIME2 v2021.8 implementation of cutadapt v3.4, python v3.8.10, R v4.0.5, and DADA2 v1.18.0 [58–61]. Default parameters were used, reads were trimmed to first instance where the 25^th^ quartile of summarized quality scores was <30, and taxonomy was classified using the SILVA 138 SSU database [62]. QIIME2 objects were imported into R via the qiime2r package v.099 [63]. ITS reads were processed by first quality filtering, trimming, and merging paired reads using the default parameters for AMPtk v1.5.5 [64], then DADA2 v1.26.0 was used to infer ASVs, remove chimeras, and assign taxonomy via the UNITE v9.0 database. All downstream operations were performed in R v.4.2.3 using phyloseq v.1.42 [65] and tidyverse v1.3.2 [66]. Rarefaction curves illustrated sufficient sequencing depth in all datasets, thus the data were not rarefied or further processed. To compare data between datasets, abundance values were summed by genus, and then normalized by dividing each value by the maximum value for each genus and for each site. These normalized abundance values were then plotted across time with ggplot2 v. 3.4.4.

### Chemical extractions

Ten 5 mm plugs were collected from culture plates and placed in a 2 ml microtube. Plugs were extracted with 750 μl ethyl acetate, sonicated for 10 min, and left at RT for 1 h. Tubes were centrifuged to pellet biomass, and extract was transferred to a new microtube and dried under vacuum at 45°C. An extraction control of sterile ISP2 agar plates was performed in parallel.

### Liquid chromatography-high-resolution mass spectrometry and liquid chromatography coupled with tandem mass spectrometry analysis

Samples were analyzed by a Ultra-High Pressure Liquid Chromatography (UHPLC) system (Dionex Ultimate 3000, ThermoFisher, USA) coupled to a HRMS (Thermo Q-Exactive Quadrupole-Orbitrap, ThermoFisher, USA) using a Heated Electrospray ionization source, using a C18 column (50 mm × 2.1 mm, 2.2 μm, Thermo Scientific Acclaim RSLC). Unless otherwise specified, the UHPLC method was as follows: 0–1 min 10% ACN + 0.1% FA, a gradient from 1 to 11 min of 10% to 99% ACN + 0.1% FA, 11–14.5 min of 99% ACN + 0.1% FA and re-equilibration of the column back into 10% ACN + 0.1% FA from 14.5 to 18 min, injection volume of 5 μl, flow rate of 0.4 ml/min, and column oven at 35°C. The full MS1 scan was performed in positive mode, resolution of 35 000 full width at half-maximum (FWHM), automatic gain control (AGC) target of 1 × 10e6 ions and a maximum ion injection time (IT) of 100 ms, mass range from m/z 200 to 2000. MS/MS analysis was acquired simultaneously using a data-dependent Top5 method at a resolution of 17 500 FWHM, AGC target of 1 × 10e5 ions, and maximum ion IT of 50 ms, using an isolation window of 3 m/z and normalized collision energy (NCE) of 20, 30, and 45. Cone spray voltage was 3.5 kV.

### Extraction and bioactivity-guided isolation of rhamnolipid methyl esters from *P. kirstenboschensis* F3

A 4 L culture of *P. kirstenboschensis F3* was grown for 5 days at 30°C on 150 × 50 mm plates containing 40 ml of ISP2 agar medium. Agar was chopped and extracted twice in 1:1 ethyl acetate, sonicated for 10 min, and left at RT for 12 to 18 h. Extract was filtered using a Whatman 114 V filter. The filtrate was dried with anhydrous sodium sulfate and concentrated using a rotary evaporator. The crude extract was partially purified by solid phase extraction (SPE) using a C-18 Sep-Pak Vac 35 cc, 10 g cartridge (Waters). Extract was loaded onto the column in 50% methanol (MeOH) and eluted in increasing concentrations of 50%, 60%, and 100% MeOH. The fractions were tested for activity against *P. omphalodes* and dried. Activity assays revealed the active compound to be in the 100% MeOH fraction. The 100% MeOH fraction was injected onto a C-18 column (250 mm × 4.6 mm, 5.0 μm, 120 Å; Thermo Scientific Acclaim RSLC) and analyzed *via* LC–MS/MS (1.0 ml/min flow) to optimize peak separation prior to semi-preparative HPLC purification.

Semi-preparative HPLC was run on a UHPLC system (Dionex UltiMate 3000, Thermo Fisher) with a C-18 column (250 mm × 10 mm, 5.0 μm, 120 Å; Thermo Scientific Acclaim RSLC). The 100% MeOH SPE fraction was dried and resuspended in 90% MeOH. HPLC was run at 5.9 ml/min flow with water (Solvent A) and acetonitrile (Solvent B) using the following gradient: 0–1 min 90% B, a gradient from 1 to 11 min of 90% to 99% B, 11–15 min 99% B, a gradient from 15 to 15.5 min of 99% to 90% B, and re-equilibration of the column at 90% from 15.5 to 19.5 min. Multiple injections of 300 μl were run and fractions were pooled. Fractions were collected every 10 s from 8 to 15.5 min and dried in a SpeedVac (SPD-1010–115, Thermo Fisher). Rhamnolipid methyl esters did not exhibit a strong UV absorbance profile, and bioactivity-guided fractionation was paired with LC–MS/MS detection. Fractions 13–18 (10 to 11 min) exhibited activity and contained ions [M + NH^4^]^+^ = 738.4995 and [M + Na]^+^ = 743.4558. Fractions 35–39 (13.7 to 14.5 min) also exhibited activity and contained ions [M + NH^4^]^+^ = 766.5311 and [M + Na]^+^ = 771.4865, corresponding to RLME B.

### Structural characterization of rhamnolipid methyl esters A-C

The 1D and 2D NMR spectra of RLME A, including ^1^H NMR, ^1^H–^1^H COSY, ^1^H–^13^C HSQC, and ^1^H–^13^C HMBC spectra, were acquired, respectively, on a Bruker Avance 900 NMR spectrometer (900 MHz for ^1^H and 225 MHz for ^13^C) equipped with a cryoprobe. For the NMR test, the sample was dissolved in DMSO-*d_6_* (Cambridge Isotope Laboratories, Inc.). Data were collected and reported as follows: chemical shift, integration multiplicity (s, singlet; d, doublet; t, triplet; m, multiplet), and coupling constant. Chemical shifts were reported using the DMSO-*d_6_* resonance as the internal standard for ^1^H-NMR DMSO-*d_6_*: *δ* = 2.50 *p.p.m.* and ^13^C-NMR DMSO-*d_6_*: *δ* = 39.5 *p.p.m.*

RLME A was isolated as a white amorphous solid. The length of the two lipids chains was revealed by MS/MS analyses. The presence of two rhamnose groups was separately supported by their COSY correlations. The stereochemistry of these two rhamnose moieties was indicated by their proton–proton coupling constants. The connection between these two sugar rings was assigned as C-2′-O-C-1″, based on ^3^*J*-HMBC correlations from H-2′ (*δ*_H_ 3.60) to C-1″ (*δ*_C_ 101.7) and from H-1″ (*δ*_H_ 4.78) to C-2′ (*δ*_C_ 98.3). The di-rhamnose was linked to C-14, following confirmed by ^3^*J*-HMBC correlations from H-14 (*δ*_H_ 3.86) to C-1′ (*δ*_C_ 98.3) and from H-1′ (*δ*_H_ 4.67) to C-2′ (*δ*_C_ 73.5). The configurations of C-3 and C-14 were both assigned as R based on the biosynthetic pathway.

RLME B was isolated as a white amorphous solid. RLME C was not isolated. The structures of RLME B and C were determined by MS/MS analyses in comparison to MS/MS spectra obtained for RLME A.

### Stable isotope labeling experiments with D_3_-L-methionine

*P. kirstenboschensis* F3 was grown in a MM, containing 12.8 g/l Na_2_HPO_4_, 3.0 g/l KH_2_PO_4_, 0.5 g/l NaCl, 1.0 g/l NH_4_Cl, 4 g/l glucose, 2 mM MgSO_4_ solution, 0.1 mM CaCl_2_ solution, 10 ml/l of RPMI 1640 B vitamins mixture (Sigma) and 150 mg/l of each of the 20 amino acids, supplemented with or without (control) 1 mg/ml D_3_-labeled methionine. About 2 ml liquid cultures were adjusted to an OD 600 of 0.05 using *P. kirstenboschensis F3* overnight cultures in the control medium and incubated at 30°C with shaking at 200 rpm for 36 h. Cultures were extracted in 1:1 ethyl acetate. The ethyl acetate layer was dried down and resuspended in 300 μl methanol, which was transferred to an LC–MS vial with a glass insert. Samples were analyzed by UHPLC–MS/MS as described above.

### *P. kirstenboschensis* F3 genome sequencing, assembly, and annotation

Genomic DNA was extracted from *P. kirstenboschensis* F3 that was grown in ISP2 for 18 h, using a modified phenol/chloroform extraction, where RNase A was added during the lysozyme incubation step [67]. DNA quality was confirmed using gel electrophoresis, Bioanalyzer, a NanoDrop One UV–Vis spectrophotometer, and a Qubit fluorometer (Invitrogen Qubit DNA-HS assay kit, Q32851). Genomic DNA was run through the UCB QB3 PacBio Sequel II sequencing pipeline. Raw reads were partitioned using seqtk, and the genome was assembled de novo with Flye into three contigs at 989x coverage. The draft genome was annotated using the NCBI Prokaryotic Genome Annotation Pipeline [68]. 

Species identification was determined by ANI analysis using the Kostas lab ANI calculator [69] in comparison to the NCBI reference genome for *P. kirstenboschensis* (GenBank accession number GCA_904848585.1). *P. kirstenboschensis* was among a list of *Paraburkholderia* species that had a > 97% identity match from BLAST searches using 16S rRNA, *gyrB*, *recA*, *rpoB*, and *trpB* DNA sequences from strain F3. Only *P. kirstenboschensis* had an ANI of >95%, an acceptable cutoff for bacterial species identification [70], with a resulting 96.83% ANI.

### Construction of *rhl* knockout strains and genetic complementation

Chromosomal gene deletions in *P. kirstenboschensis* F3 were generated *via* double allelic exchange mediated by a pEXG2-based suicide vector [71]. For each gene deletion strain, a separate suicide vector was constructed containing an insert comprised of a ~ 500 bp upstream homology region and a ~ 500 bp downstream homology region from the target gene. Primers consisted of a (19–21 nt) binding region and a 20-nt 5′ overhang complementary to the other fragment (backbone or insert). Primers used to amplify the upstream and downstream regions of the target genes are listed in Table S2. The insert and PCR-amplified backbone were assembled via Gibson assembly [72], and transformed into *E. coli* DH5α (New England Biolabs) for plasmid selection. Single colonies were picked from LB gentamicin selection plates, and plasmids recovered using a miniprep kit (Bioneer AccuPrep Plasmid Mini Extraction Kit). Recovered plasmids were sequenced at the UC Berkeley DNA Sequencing Facility to confirm no mutations in the homology region. Plasmids were electroporated into *P. kirstenboschensis* F3, and merodiploids were selected on LB gentamicin plates. Single colonies were streaked onto NSLB+15% (w/v) sucrose plates to counterselect for double-crossover events. True recombinants (gentamicin sensitive and sucrose resistant) were resolved from merodiploids (gentamicin resistant and sucrose sensitive) based on the loss of two vector-associated markers, gentamicin resistance (*aaC1*) and sucrose sensitivity (*sacB*). Plasmids for genetic complementation were constructed with Gibson assembly of a PCR-amplified gene into the pBBR1-MCS5 vector backbone [73].

### Swarming motility assay and atomized oil assay

Seed cultures of each strain were grown in ISP2 liquid medium overnight at 30°C. After 16–20 h of growth, cultures were then subcultured 1:20 into fresh liquid medium and grown to an OD of ~0.6. Cells were pelleted, washed twice with PBS, and resuspended to an OD of 0.5 in PBS; 5 μl of each cell suspension was spotted at the center of a plate (ISP2 with 0.25% agar for swarming motility assays, or modified M9 MM [20 mM ammonium chloride, 12 mM sodium phosphate dibasic, 22 mM potassium phosphate monobasic, 8.6 mM sodium chloride, 1 mM magnesium sulfate, 1 mM calcium chloride, 11 mM dextrose, 0.5% casamino acids] with 0.5% agar for atomized oil assays). Assays were performed with five replicates per strain. Plates were incubated for 3 days at RT, then imaged using an iPhone 12S camera. For atomized oil assays, plates were sprayed with a thin layer of light mineral oil (Fisher Scientific) and imaged using an iPhone 12S camera with an oblique light source. Motility diameters and surfactant zone diameters were measured by randomly choosing two orthogonal lines and obtaining the average. Data were analyzed for statistical significance using a one-way ANOVA and post hoc Tukey’s test (*P* < .05).

### Race tube assay

About 15 ml PyOM agar (10 g/l ground PyOM, 1× Vogel’s salts [57], 1% Bacto agar) was added to glass race tubes (circumference 5 cm, length 38 cm, bent upward at 5 cm from each end) and allowed to solidify and cool in a biosafety cabinet for 1 h. *Paraburkholderia* strains were cultured in liquid ISP2 for 20 h at 30°C, washed twice with PBS, and adjusted to OD 3.0 in PBS. About10 μL of the OD 3.0 cell suspension was spotted on PyOM agar at one end of the race tube; 10 μL PBS was spotted as a negative control. Twenty-four hours after *Paraburkholderia* inoculation, *P. omphalodes* 1672 was inoculated from a 4-day old culture on VMM agar. An agar transfer tube (5 mm diameter) was used to punch uniform circular samples of *P. omphalodes*, and sterile tweezers were used to separate mycelium from the agar. *P. omphalodes* was placed on PyOM agar at the opposite end of either *Paraburkholderia* or PBS. Foam stoppers were inserted at both ends of each race tube to maintain sterility. Race tubes were incubated at RT, within a box containing three beakers each filled with 250 ml water to prevent drying. Measurements were taken at the farthest reaching point of *P. omphalodes* mycelia at each time point. Images were acquired at 18.6× magnification using a Zeiss Axio Zoom V16 microscope equipped with a Tucsen 16-bit camera (Dhyana 400BSI). Statistical significance was determined using Welch’s *t*-test between two groups.

### Polycyclic aromatic hydrocarbon solubilization

About 0.25 mg of RLME A or rhamnolipids (95%, di-rhamnolipid dominant, Sigma-Aldrich) was aliquoted into 2-ml scintillation vials. Excess (~10 mg) of either naphthalene, phenanthrene, or benzo[*a*]pyrene was measured into vials containing RLME A, rhamnolipids, or nothing (base solubilization controls). Each treatment was tested in triplicate, except for the water and rhamnolipids conditions for phenanthrene, which were tested in duplicate; 500 μl of sterile water with 0.01 M sodium azide (to inhibit microbial growth) was added to each vial, which were then sonicated for 10 min. The mixtures were pipetted into 1.5-ml microcentrifuge tubes and centrifuged for 20 min at 15000 × *g* to pellet the solids. Supernatants were transferred to clean glass vials and dried by SpeedVac. Samples were resuspended in 2× dichloromethane (DCM), diluted up to 2-fold, and measured using a UV–Vis spectrophotometer (Thermo Scientific Genesys 10S) at 228 nm (naphthalene), 254 nm (phenanthrene), and 295 nm (benzo[*a*]pyrene). A standard curve was constructed from solutions of naphthalene (2.5–15 mg/l), phenanthrene (0.5–2.5 mg/l), or benzo[*a*]pyrene (0.55–2.75 mg/l) in DCM (Fig. S17). Beer’s law was used to determine concentrations from absorbance readings using the standard curve. Statistical significance was determined using a one-way ANOVA and post hoc Tukey’s test (*P* < .05).

### RhlM ligand docking and molecular dynamics

A protein model for RhlM was generated using AlphaFold through the LatchBio interface. Protein structure alignments were performed in Maestro. RhlM was aligned with Ma MTase (PDB ID: 4a2n), and the SAH ligand was extracted from Ma MTase and docked into the RhlM AlphaFold model using Glide. This RhlM-SAH structure was then used to define the receptor grid for docking SAM. The RhlM-SAM structure was used to dock Rha-Rha-C_14_-C_10_ (RL, compound 5) within a defined receptor grid and with an applied positional constraint of 4 Å between the RL carboxylate O and the SAM methyl C. Molecular dynamics simulations were performed using Desmond with a modeled lipid membrane environment (DPPC), preequilibrated at 325 K. Residues 4–24, 35–55, 76–94, and 144–163 were manually selected for inclusion in membrane-bound region. Multiple sequence alignments were performed using Clustal Omega. Structural alignments of RhlM with *M. acetivorans* MTase (PDB: 4a2n) and *T. castaneum* ICMT (PDB: 5vg9) were performed using ChimeraX [74]*.*

### Sequence similarity and genome neighborhood analysis

A colored SSN was generated using the EFI-EST SSN tool for the ICMT family using the Pfam PF04140 with the sequence for RhlM added [31]. An alignment score of 35 and sequence length cutoffs of no shorter than 150 and no longer than 330 amino acids was used. This SSN was visualized and analyzed using Cytoscape [75]. The EFI genome neighborhood tool (GNT) was then applied to this SSN using standard parameters to calculate the Genome Neighborhood Diagrams (GND). Operons within the cluster containing RhlM (Cluster 14) were visually inspected in the GND explorer.

### Phylogenetic tree construction 

Species trees were constructed using KBase [76] and visualized and annotated using iTOL version 6.8.1 [77].

#### Cluster 14 species tree

For each species represented within the SSN Cluster 14, one genome was curated from the NCBI database and input into Kbase by the RefSeq ID. In cases where multiple genomes were available, the reference genome was used. In cases where no complete genome was available (e.g. for sequences originating from metagenome datasets), a representative genome was omitted. Due to these omissions as well as repeat instances of the same species represented in the SSN cluster, the original 92 members of Cluster 14 were reduced to 53 for tree construction. An outgroup (*M. acetivorans* C2A) was included for tree calculation and then manually removed to faciliate visualization. GNDs were downloaded from the EFI-GNT analysis and recolored.

#### Burkholderia sensu lato species tree

Representative genomes for each species listed in NCBI under the genera *Burkholderia*, *Caballeronia*, *Mycetohabitans*, *Paraburkholderia*, *Pararobbsia*, *Robbsia*, and *Trinickia* were curated and input into KBase by the RefSeq ID. Reference genomes were used when available, otherwise, one genome was randomly selected. Of the 184 total species, 18 were not found in the KBase database and were omitted from the tree. A BLAST search was performed across all sequences in the NCBI database in the above seven genera, using *P. kirstenboschensis* F3 protein sequences RhlA and RhlM in two independent queries. The presence of the rhamnolipid BGC and *rhlM* was verified by examining the genomic context. For each species, the rhamnolipid BGC and/or RhlM was considered present if at least one genome produced a match from the BLAST query.

## Supplementary Material

Liu_RLME_SI_V4_Revised_V5_changesaccepted_wrae022

## Data Availability

*P. kirstenboschensis* F3 genome data were deposited to NCBI (accession number CP136511-CP136513).
