# Supplementary material for: Surface-active antibiotic production as a multifunctional adaptation for postfire microorganisms
Source: ISME J. 2024 Feb 5;18(1):wrae022. doi: 10.1093/ismejo/wrae022 (PMC11069360; doi:10.1093/ismejo/wrae022)
Supplement: Liu_RLME_SI_V4_Revised_V5_changesaccepted_wrae022 [file liu_rlme_si_v4_revised_v5_changesaccepted_wrae022.pdf]

Supplementary Information

Surface-active antibiotic production as a multifunctional adaptation for postfire microorganisms

Mira D. Liu[1], Yongle Du[2], Sara K. Koupaei[3], Nicole R. Kim[3], Monika S. Fischer[3], Wenjun Zhang[2], and Matthew F. Traxler[3]*

[1]Department of Chemistry, University of California, Berkeley, Berkeley 94720, USA.
[2]Department of Chemical and Biomolecular Engineering, University of California, Berkeley, Berkeley 94720, USA.
[3]Department of Plant and Microbial Biology, University of California, Berkeley, Berkeley 94720, USA.
*Corresponding author: Matthew F. Traxler

Contents

Supplementary Tables 3

Supplementary Table 1. Strains used in this study
Supplementary Table 2. Oligonucleotide sequences used for plasmid construction and sequencing
Supplementary Table 3. Strains used in antifungal screen
Supplementary Table 4. NMR data of Compound 720 (RLME A) in DMSO-d_6

Supplementary Table 5. Gene identifiers for *rhl* biosynthetic pathway in *P. kirstenboschensis* F3
Supplementary Table 6. Post-fire fungi used in *P. kirstenboschensis* F3 inhibition assays
Supplementary Table 7. Post-fire bacteria used in *P. kirstenboschensis* F3 inhibition assays
Supplementary Table 8. Protein structural alignment comparisons
Supplementary Table 9. Prevalence of RLMEs among burned soil *Paraburkholderia* isolates

Supplementary Figures 15

Supplementary Figure 1. Abundance of *Pyronema* and *Paraburkholderia* in burned soils over time
Supplementary Figure 2. Average nucleotide identity analysis of *Paraburkholderia kirstenboschensis* F3 genome
Supplementary Figure 3. ^{1}H NMR spectrum of rhamnolipid methyl ester A, DMSO-d_6 at 900 MHz
Supplementary Figure 4. ^{1}H-^{1}H COSY spectrum of rhamnolipid methyl ester A, DMSO-d_6 at 900 MHz
Supplementary Figure 5. ^{1}H-^{13}C HSQC spectrum of rhamnolipid methyl ester A, DMSO-d_6 at 900 MHz
Supplementary Figure 6. ^{1}H-^{13}C HMBC spectrum of rhamnolipid methyl ester A, DMSO-d_6 at 900 MHz
Supplementary Figure 7. HRMS/MS spectra for rhamnolipid methyl ester B and C
Supplementary Figure 8. Relative abundances of rhamnolipid methyl ester A-C
Supplementary Figure 9. RLME A and B inhibition of *Pyronema omphalodes* 1672
Supplementary Figure 10. Extracted ion chromatograms for *P. kirstenboschensis* F3 strains
Supplementary Figure 11. Heatmap of putative rhl biosynthetic intermediates
Supplementary Figure 12. Stable isotope labeling using D_3-Methionine
Supplementary Figure 13. RLME A, RLME B, and RL inhibition of post-fire *Amycolatopsis* spp. isolates
Supplementary Figure 14. Quantification of swarming motility and surfactant fronts for *P. kirstenboschensis* F3 WT, *rhl* mutants, and genetic complementation strains
Supplementary Figure 15. Predicted pathways for toluene degradation in *P. kirstenboschensis* F3
Supplementary Figure 16. Predicted pathways for benzoate degradation in *P. kirstenboschensis* F3
Supplementary Figure 17. Standard curves for PAH solubilization experiments

Supplementary Figure 18. *P. kirstenboschensis* motility in race tube microcosms
Supplementary Figure 19. AlphaFold structure of RhlM colored by pLDDT score
Supplementary Figure 20. RhlM interactions with S-adenosyl methionine
Supplementary Figure 21. RhlM interactions with RL (compound 5)
Supplementary Figure 22. Phylogenetic tree of SSN Cluster 14 representatives and genomic context diagrams

Supplementary Tables

Table S1. Strains used in this study.

Strain ID	Species/Strain Name	Relevant Genotype	Source
F3	*Paraburkholderia kirstenboschensis* F3	Wildtype	This study
ML59	*Paraburkholderia kirstenboschensis* F3	Δ*rhlM*	This study
ML65	*Paraburkholderia kirstenboschensis* F3	Δ*rhlB*	This study
ML91	*Paraburkholderia kirstenboschensis* F3	Δ*rhlA*	This study
ML77	*Paraburkholderia kirstenboschensis* F3	Δ*rhlM* + pBBR-MCS5-rhlM	This study
ML84	*Paraburkholderia kirstenboschensis* F3	Δ*rhlB* + pBBR-MCS5-rhlB	This study
ML137	*Paraburkholderia kirstenboschensis* F3	Δ*rhlA* + pBBR-MCS5-rhlA	This study
C1	*Paraburkholderia kirstenboschensis* C2	Wildtype	This study
C2	*Paraburkholderia strydomiana* C1	Wildtype	This study
A3-S	*Paraburkholderia kirstenboschensis* A3-S	Wildtype	This study
D1	*Paraburkholderia kirstenboschensis* D1	Wildtype	This study
D6-S	*Paraburkholderia kirstenboschensis* D6-S	Wildtype	This study
G6	*Paraburkholderia kirstenboschensis* G6	Wildtype	This study
G7-S	*Paraburkholderia kirstenboschensis* G7-S	Wildtype	This study
1672	*Pyronema omphalodes*	Wildtype	Tom Bruns

Wild soil isolates were identified by closest 16S rRNA match on NCBI at the time of deposition to the collection. All *P. kirstenboschensis* F3 knockout and complementation strains were generated using primers in Table S2.

Table S2. Oligonucleotide sequences used for plasmid construction and sequencing.

Name	Sequence (5' → 3')
16S-27F	AGAGTTTGATCCTGGCTCAG
16S-1492-R	GGTTACCTTGTTACGSCTT
**Primers for gene deletion**	
RhlM-Up-F	aagcttctgcaggtcgactcCGATGCTCGCCTATGTGACCC
RhlM-Up-R	gacctcaatagagCGTGTGGTAGATCGTCATTTTCAG
RhlM-Down-F	atgacgatctaccacacgCTCTATTGAGGTCCGCGCG
RhlM-Down-R	gagcccggggatcctctaAGCACGCCTTTCTCGATCATTTC
pEXG2-RhlM-Gibson-F	aaatgatcgagaaaggcgtgctTAGAGGATCCCCGGGCTCG
pEXG2-RhlM-Gibson-R	ggtcacataggcgagcatcgGAGTCGACCTGCAGAAGCTTGC
RhlM-Seq-F	TCATTGCTCGCCCTGGCG
RhlM-Seq-R	GGCCGAGGATACGGCTTG
pEXG2-seq-F	tgttgcatgggcataaagttg
pEXG2-seq-R	tcaacgacaggagcacgatc
RhlB-Up-F	ttccacacattatacgagccggaagcataaatgtaaagcaAAGCGTCCTCCTGGGCGAAG
RhlB-Up-R	gttcaggcgacTGCGGTGATGACGATTTGTGC
RhlB-Down-F	aatcgtcatcaccgcaGTCGCCTGAACGAATCCGG
RhlB-Down-R	taaggtaccgaattcgagctcgagcccggggatcctctagCTGGTCGATGATCCAGCCGC
RhlB-Seq-F	GTCTGGCTCGTCTACTGG
RhlB-Seq-R	AAAGTGCTGCGCATGCGG
pEXG2-RhlB-Gibson-F	gcggctggatcatcgaccagCTAGAGGATCCCCGGGCTC
pEXG2-RhlB-Gibson-R	cttcgcccaggaggacgcttTGCTTTACATTTATGCTTCCGGC
RhlA-Up-F	caagcttctgcaggtcgactcGTCTTGCGGCCTAGGTGTC
RhlA-Up-R	ccgaaatcggtgttTTCGACGGACATAGAGCCCC
RhlA-Down-F	tctatgtccgtcgaaAACACCGATTTCGGAGGCTG
RhlA-Down-R	cgagcccggggatcctctaTACGGGTCGCCGAAATACTCG
RhlA-Seq-F	ACATACCCGCGACCGGC
RhlA-Seq-R	TCG CGC GGA CCT CAA TAG
pEXG2-RhlA-Gibson-F	cgagtatttcggcgacccgtaTAGAGGATCCCCGGGCTCG
pEXG2-RhlA-Gibson-R	gacacctaggccgcaagacGAGTCGACCTGCAGAAGCTTGC
**Primers for genetic complementation constructs**	
RhlM-F	aattcgatatcaagcttatcgGAGTCGCGCGGACCTCAATAG
RhlM-R	ccctcgaggtcgacggtatTTGAACACCGATTTCGGAGGCTG
BB-RhlM-F	gcctccgaaatcggtgttcaaATACCGTCGACCTCGAGGG
BB-RhlM-R	attgaggtccgcgcgactcCGATAAGCTTGATATCGAATTCCTGC
RhlB-F	aattcgatatcaagcttatcgTCAGGCGACCGAACGCGTG
RhlB-R	cccctcgaggtcgacggtatATTGAGGTCCGCGCGACTC
BB-RhlB-F	tgagtcgcgcggacctcaatATACCGTCGACCTCGAGGG
BB-RhlB-R	cacgcgttcggtcgcctgaCGATAAGCTTGATATCGAATTCCTGC
RhlA-F	cccctcgaggtcgacggtatCATCCACCGTAACATCCTGG
RhlA-R	gcaggaattcgatatcaagcttatcgCAAGCGTGTGGTAGATCGTC
BB-RhlA-F	gacgatctaccacacgcttgCGATAAGCTTGATATCGAATTCCTGC
BB-RhlA-R	ccaggatgttacggtggatgATACCGTCGACCTCGAGGGG
pBBR1MCS5-Seq-R	CAGGAAACAGCTATGACC
pBBR1MCS5-Seq-F	TGTAAAACGACGGCCAGT

F = forward, R = reverse. Lowercase letters represent Gibson homology.

Table S3. Preliminary antifungal screen results.

Strain	16S BLAST Result	Source			Plug Assay Result		
		Plot	Depth	Collection Date	ISP2	PyOM	ISP2 vs. PyOM comparison
A3-S	*Paraburkholderia kirstenboschensis*	321E	3-6 cm	29-Oct-18	Y	N.T.	N/A
B7	*Streptomyces fumigatiscleroticus*	321E	3-6 cm	27-Nov-18	M	N.T.	N/A
B8	*Caballeronia udeis*	321E	10 cm	16-Oct-18	N	N.T.	N/A
C1	*Paraburkholderia* sp.	321E	3-6 cm	29-Oct-18	Y	Y	P
C2	*Paraburkholderia* sp.	240	10 cm	12-Oct-18	Y	Y	P
C5	*Paraburkholderia* sp.	321E	3-6 cm	27-Nov-18	Y	Y	I
C8	*Streptomyces phaeogriseichromatogenes*	321E	3-6 cm	27-Nov-18	M	N.T.	N/A
C9	*Amycolatopsis* sp.	321E	0-3 cm	29-Oct-18	M	N.T.	N/A
D1	*Paraburkholderia* sp.	240	10 cm	12-Oct-18	Y	Y	E
D5	*Pseudomonas* sp.	321E	0-3 cm	27-Nov-18	N	N.T.	N/A
D6-S	*Paraburkholderia* sp.	321E	0-3 cm	29-Oct-18	Y	Y	P
D6-W	*Streptomyces* sp.	321E	0-3 cm	29-Oct-18	Y	N	I
D7	*Paraburkholderia caledonica*	321E	0-3 cm	20-Nov-18	Y	Y	E
E1	*Streptomyces* sp.	321E	3-6 cm	20-Nov-18	M	M	E
E3	*Caballeronia udeis*	321E	10 cm	16-Oct-18	N	N	N/A
E4	*Agrobacterium rhizogenes*	321E	3-6 cm	20-Nov-18	M	N.T.	N/A
E5	*Streptomyces adamanensis*	321E	3-6 cm	27-Nov-18	M	N	I
E6	*Paenibacillus tundrae*	321E	3-6 cm	20-Nov-18	Y	N.T.	N/A
E7	*Paenibacillus tundrae*	321E	3-6 cm	20-Nov-18	M	N.T.	N/A
E8	No match	321E	3-6 cm	20-Nov-18	Y	N	I
E9	*Paenibacillus rigui*	321E	3-6 cm	20-Nov-18	M	N.T.	N/A
F2	No match	321E	0-3 cm	27-Nov-18	N	N	N/A
F3	*Paraburkholderia* sp.	321E	3-6 cm	20-Nov-18	Y	Y	P
F6	*Paraburkholderia* sp.	321E	3-6 cm	29-Oct-18	N	N	N/A

Strain	16S BLAST Result	Source			Plug Assay Result		
		Plot	Depth	Collection Date	ISP2	PyOM	ISP2 vs. PyOM comparison
F7	*Paraburkholderia caledonica*	321E	10 cm	16-Oct-18	Y	Y	E
F8	*Paraburkholderia* sp.	321E	3-6 cm	27-Nov-18	Y	Y	E
F9	*Kribbella soli*	321E	0-3 cm	27-Nov-18	N	N	N/A
G1	No match	321E	0-3 cm	27-Nov-18	M	N	I
G6	*Paraburkholderia* sp.	321E	10 cm	16-Oct-18	Y	Y	P
G7-S	*Paraburkholderia caledonica*	321E	3-6 cm	27-Nov-18	Y	Y	E
G8	*Paraburkholderia caledonica*	321E	3-6 cm	27-Nov-18	Y	Y	E
G9	*Caballeronia udeis*	321E	10 cm	16-Oct-18	N	N.T.	N/A
H9	*Streptomyces* sp.	321E	3-6 cm	29-Oct-18	M	N	I
H9-YW	*Streptomyces brevispora*	321E	3-6 cm	29-Oct-18	Y	N.T.	N/A
I2	*Paraburkholderia* sp.	321E	3-6 cm	29-Oct-18	Y	Y	E
I3	No match	321E	0-3 cm	29-Oct-18	M	N	I
I6	No match	321E	3-6 cm	29-Oct-18	M	N	I
ICB3-9	*Amycolatopsis* sp.	ICB	10 cm	15-Nov-18	N	N	N/A

Y = Zone of inhibition observed
N = No zone of inhibition observed
M = Moderate zone of inhibition observed
N.T. = Not tested

E = Equal zone sizes from ISP2 and PyOM cultures
I = ISP2 culture produced larger zone of inhibition
P = PyOM culture produced larger zone of inhibition
N/A = Not applicable

Table S4. NMR Data of Compound 720 (Rhamnolipid methyl ester A) in DMSO-d_6.

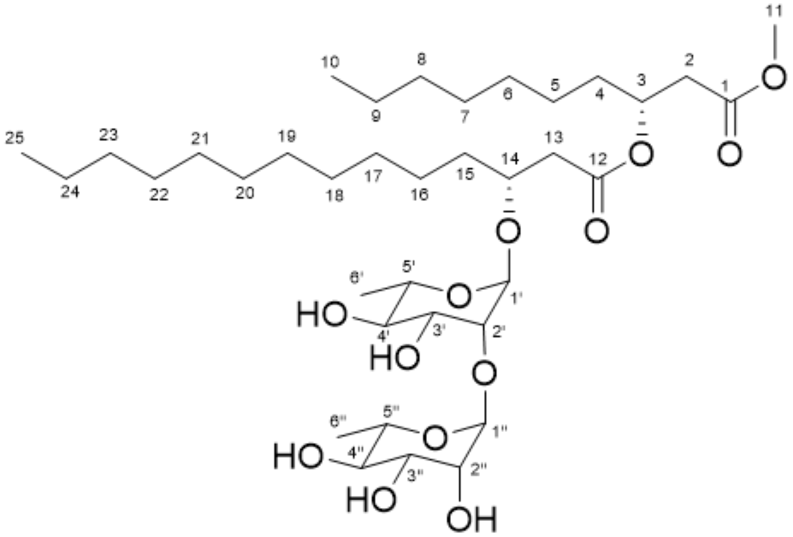

Position	δ_H (*J* in Hz)	δ_C (C type)	Position	δ_H (*J* in Hz)	δ_C (C type)
1		170.4, C	24	0.84 d 7.0	13.7, CH_3
2	2.63 dd 15.6, 5.5	38.1, CH_2	1'	4.67 brs	98.3, CH
	2.56 dd 15.6, 7.7		2'	3.60 brs	76.5, CH
3	5.08 m	69.7, CH	3'	3.47 d 11.9	70.1, CH
4	1.54 m	33.0, CH_2	4'	3.17 dd 11.9, 11.9	71.7, CH
5	1.23 m	24.1, CH_2	5'	3.46 m	68.5, CH
6	1.23 m	28.7, CH_2	6'	1.11 d 6.2	17.5, CH_3
7	1.23 m	28.7, CH_2			
8	1.22 m	31.1, CH_2			
9	1.26 m	21.8, CH_2			
10	0.85 d 7.0	13.7, CH_3	1''	4.78 brs	101.7, CH
11	3.58 s	51.1, CH_3	2''	3.69 brs	69.9, CH
12		169.9, C	3''	3.39 dd 9.5, 3.3	70.3, CH
13	2.48 dd 15.0, 5.9	39.8, CH_2	4''	3.17 dd 9.5, 9.5	71.7, CH
	2.44 dd 15.0, 6.6		5''	3.43 m	68.4, CH
14	3.86 m	73.5, CH	6''	1.09 d 6.2	17.5, CH_3
15	1.44 m	32.6, CH_2			
16	1.23 m	24.4, CH_2			
17-22	1.23 m	28.7, CH_2			
23	1.22 m	31.1, CH_2			

Table S5. Gene identifers for *rhl* biosynthetic pathway in *P. kirstenboschensis* F3.

Gene	Locus_tag
rhlA	RW095_02155
rhlM	RW095_02150
rhlB	RW095_02145
rhlD	RW095_02140
rhlC	RW095_02135
rhlF	RW095_02130
rhlE	RW095_02125

Table S6. Post-fire fungi used in *P. kirstenboschensis* F3 inhibition assays

Strain Name	BLAST Result	Division	Family	Source	Year Isolated	Genome Sequenced	Inhibition by *P. kirstenboschensis* F3
Pyro1672	*Pyronema omphalodes*	Ascomycota	Pyronemataceae	Rim Fire (CA)	2013	Y	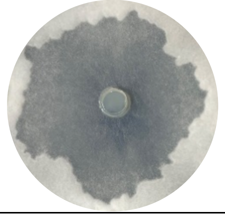
Pyro7353	*Pyronema domesticum*	Ascomycota	Pyronemataceae	Rim Fire (CA)	2013	Y	N
PyroCZU	*Pyronema sp.*	Ascomycota	Pyronemataceae	CZU Lightning Complex fires (CA)	2021	N	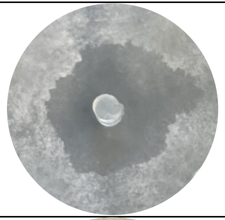
PyroSCUp	*Pyronema sp.*	Ascomycota	Pyronemataceae	SCU Lightning Complex fires (CA)	2021	N	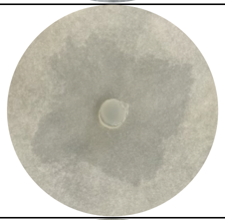
PyroSCUo	*Pyronema sp.*	Ascomycota	Pyronemataceae	SCU Lightning Complex fires (CA)	2021	N	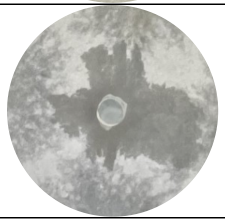

Strain Name	BLAST Result	Division	Family	Source	Year Isolated	Genome Sequenced	Inhibition by *P. kirstenboschensis* F3
PyroGlass	*Pyronema sp.*	Ascomycota	Pyronemataceae	Glass Fire (CA)	2021	N	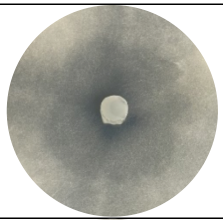
PyroOR	*Pyronema sp.*	Ascomycota	Pyronemataceae	Knoll fire (OR)	2022	N	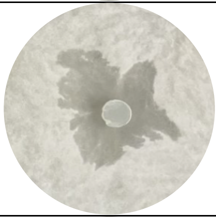
Pyro1657	*Pyronema sp.*	Ascomycota	Pyronemataceae	Rim Fire (CA)	2013	N	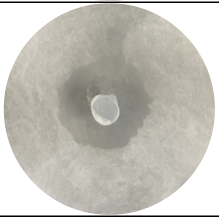
AnthBS	*Anthracobia melaloma*	Ascomycota	Pyronemataceae	Blodgett Forest (CA)	2013	N	N
Mor1602	*Morchella eximia*	Ascomycota	Morchellaceae	Rim Fire (CA)	2013	Y	N
Geo1671	*Geopyxis carbonaria*	Ascomycota	Pyronemataceae	Rim Fire (CA)	2013	Y	N
Tri1085	*Tricharina praecox*	Ascomycota	Pyronemataceae	Rim Fire (CA)	2013	Y	N
Pho1100	*Pholiota molesta*	Basidiomycota	Strophariaceae	Rim Fire (CA)	2013	Y	N
Lyo2229	*Lyophyllum atratum*	Basidiomycota	Lyophyllaceae	Rim Fire (CA)	2013	Y	N

Table S7. Post-fire bacteria used in *P. kirstenboschensis* F3 inhibition assays

Strain Name	BLAST Result	Phylum	Family	Source	Year Isolated	Genome Sequenced	Inhibition by *P. kirstenboschensis* F3
ICB3-10	*Amycolatopsis saalfeldensis*	Actinomycetota	Pseudonocardiaceae	ICB	2018	N	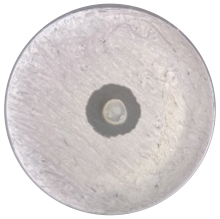
ICB3-15	*Amycolatopsis saalfeldensis*	Actinomycetota	Pseudonocardiaceae	ICB	2018	N	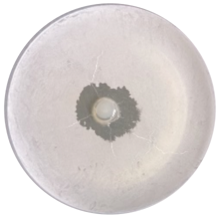
C9	*Amycolatopsis sp.*	Actinomycetota	Pseudonocardiaceae	BF	2018	N	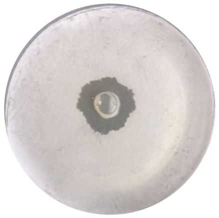
E1	*Streptomyces sp.*	Actinomycetota	Streptomycetaceae	BF	2018	Y	N.I.
E4	*Agrobacterium rhizogenes*	Proteobacteria	Rhizobiaceae	BF	2018	N	N.I.
F9	*Kribbella soli*	Actinomycetota	Kribbellaceae	BF	2018	N	N.I.
E7	*Paenibacillus sp.*	Firmicutes	Paenibacillaceae	BF	2018	N	N.I.
E9	*Paenibacillus rigui*	Firmicutes	Paenibacillaceae	BF	2018	N	N.I.

Strain Name	BLAST Result	Phylum	Family	Source	Year Isolated	Genome Sequenced	Inhibition by *P. kirstenboschensis* F3
F6	*Paraburkholderia sp.*	Proteobacteria	Burkholderiaceae	BF	2018	N	N.I.
E3	*Caballeronia sp.*	Proteobacteria	Burkholderiaceae	BF	2018	N	N.I.
BS-U/CE96c	*Paenarthrobacter nicotinovorans*	Proteobacteria	Micrococcaceae	BF	2021	N	N.I.

ICB = Illilouette Creek Basin Fire (CA)BF = Blodgett Forest (CA)
N.I. = No Inhibition

Table S8. Protein structural alignment comparisons of RhlM AlphaFold model with ICMT family crystal structures.

	PDB: 4a2n	PDB: 5vg9
Root mean square deviation (RMSD), pruned	1.118 Å	0.793 Å
RMSD, all	4.445 Å	8.275 Å
Sequence alignment score	304.1	255.7

Structural alignments performed in ChimeraX.

Table S9. Prevalence of rhamnolipid methyl esters among different *Paraburkholderia* burned soil isolates.

Strain	Species	Source			*Pyronema* inhibition	HR-MS, MS/MS	*rhlM*
		Plot	Depth	Collection date			
F3	*P. kirstenboschensis*	321E	3-6 cm	20-Nov-18	+	+	+
A3-S	*P. kirstenboschensis*	321E	3-6 cm	29-Oct-18	+	+	+
G7-S	*P. kirstenboschensis*	321E	3-6 cm	27-Nov-18	+	+	+
C2	*P. strydomiana*	240	0-10 cm	12-Oct-18	+	+	+
G6	*P. kirstenboschensis*	321E	0-10 cm	16-Oct-18	+	+	+
D1	*P. ginsengisoli*	240	0-10 cm	12-Oct-18	+	+	+
D6-S	*P. kirstenboschensis*	321E	0-3 cm	29-Oct-18	+	+	+

The prevalence of RLMEs was examined using HR-MS and MS/MS fragmentation in addition to PCR. Positive data indicate that inhibition of *Pyronema omphalodes* 1672 was observed, RLMEs were detected in culture extracts, and *rhlM* was amplified from gDNA. Plot 321E was burned on October 16, 2018, and Plot 240 was an unburned plot. Depth and Collection date refer to the collected soil samples from which the strain was subsequently isolated. Species identification is based on a BLAST search of the 16S PCR amplicon and a >97% similarity cutoff.

Supplementary Figures

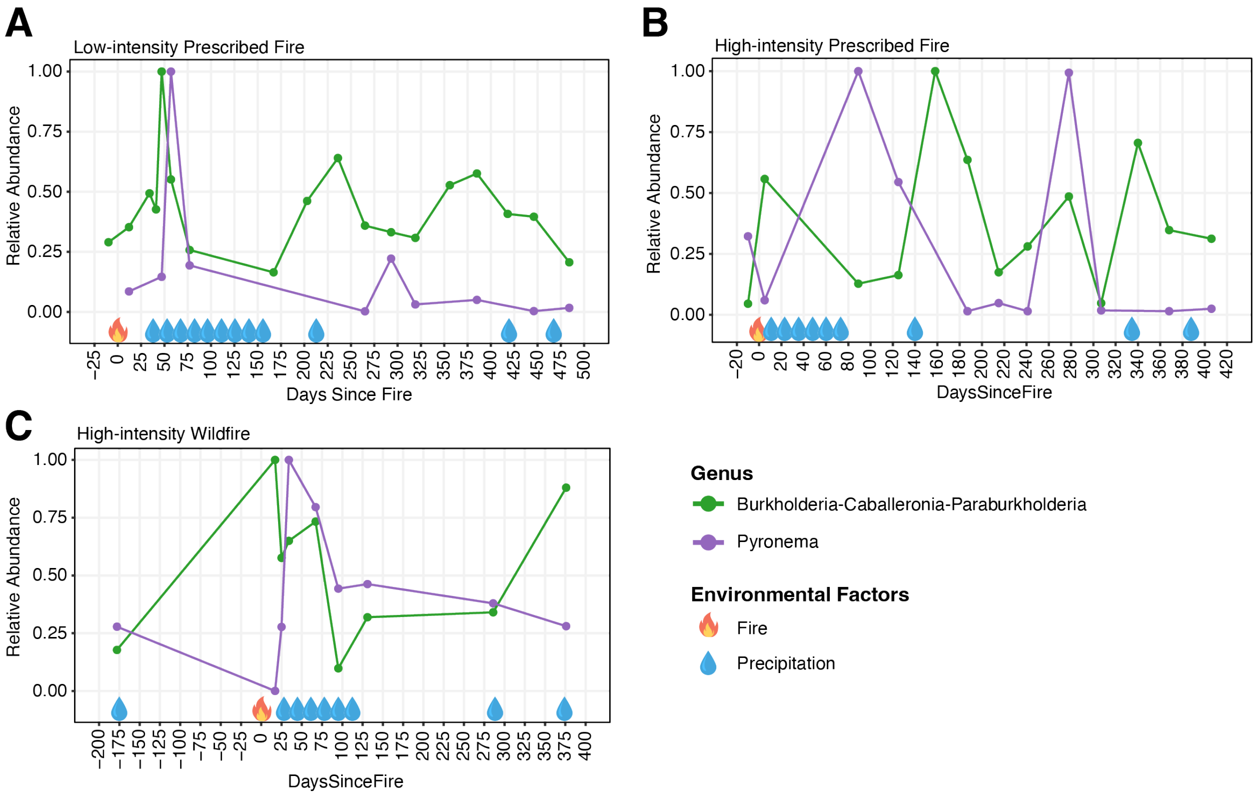

Figure S1. Abundance of Pyronema and Paraburkholderia in burned soils over time. Internal Transcribed Spacer (ITS) and 16S amplicon community sequencing datasets were re-analyzed from two previously published time-series studies; (A) low-intensity prescribed fire in a CA mixed conifer forest (Fischer & Patel, et al, 2023), (B) high-intensity prescribed fire in a CA mixed conifer forest (Fischer & Patel, et al, 2023), and (C) high-intensity wildfire in CA chaparral (Pulido-Chavez, et al, 2023). Relative abundance values were normalized to the maximum value for each genus, and each point represents the sum of all amplicon sequence variants (ASVs) per genus per site. At the level of 16S, the following genera are indistinguishable; Burkholderia, Caballeronia, and Paraburkholderia.

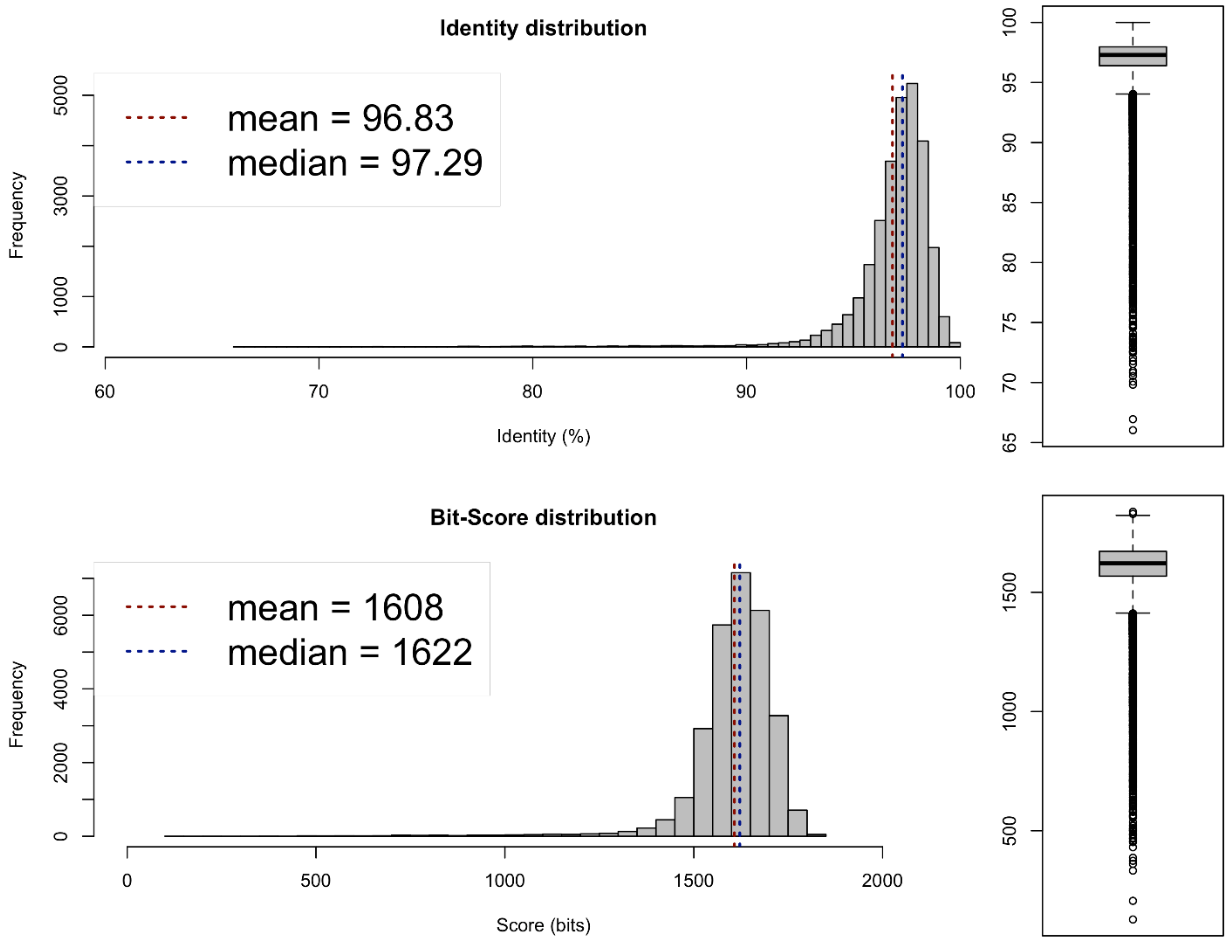

Figure S2. Average nucleotide identity (ANI) analysis of *Paraburkholderia kirstenboschensis* F3 genome.

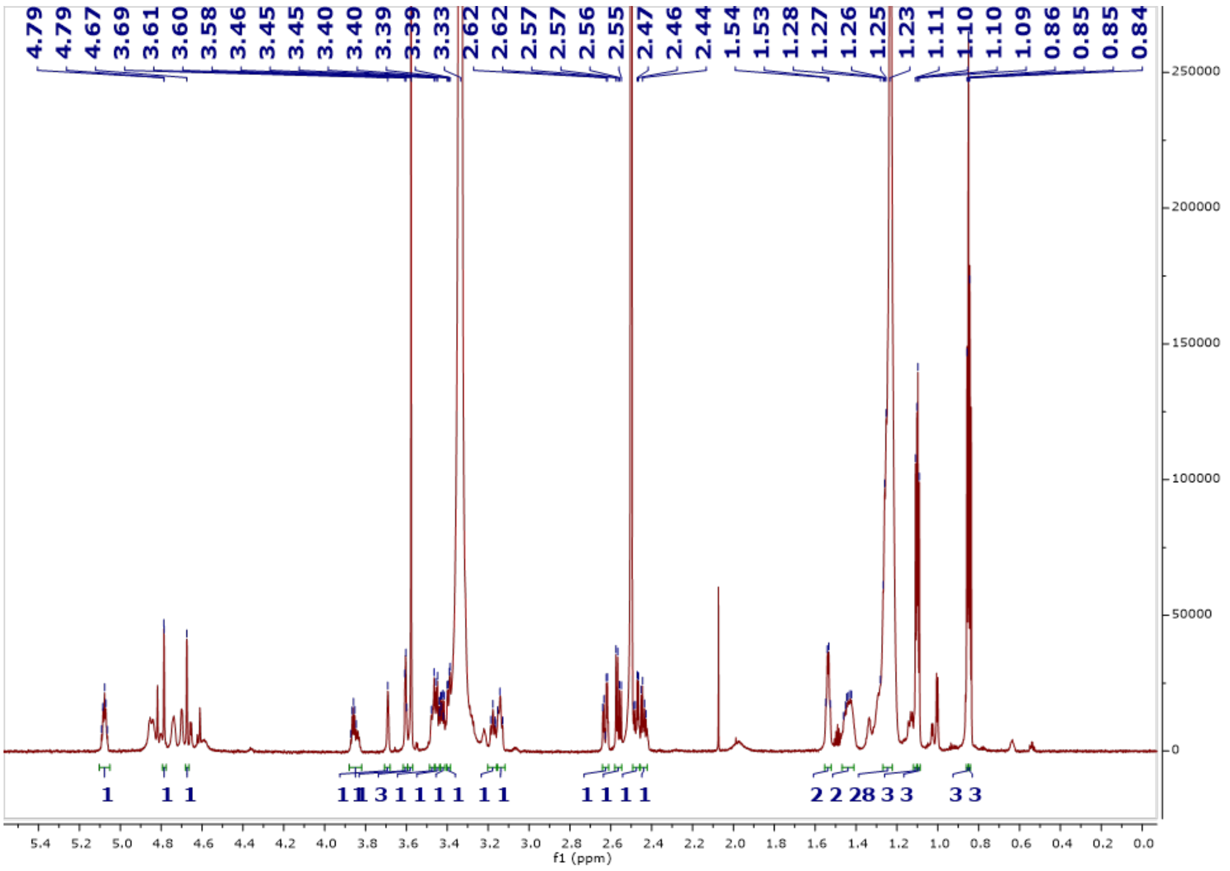

Figure S3. ^{1}H NMR spectrum of rhamnolipid methyl ester A, recorded in DMSO-d_6 at 900 MHz.

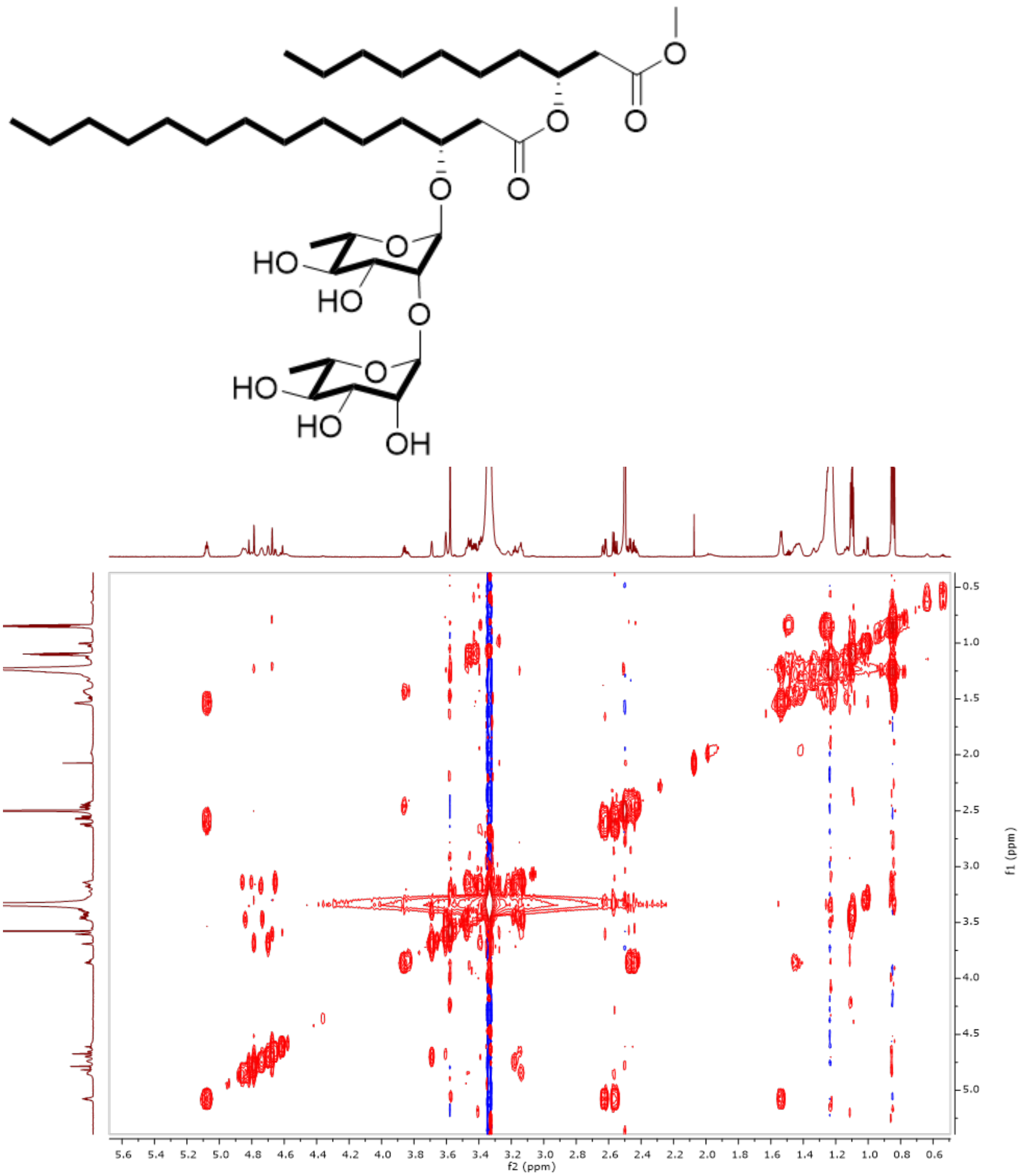

Figure S4. ^{1}H-^{1}H COSY spectrum of rhamnolipid methyl ester, recorded in DMSO-d_6 at 900 MHz.

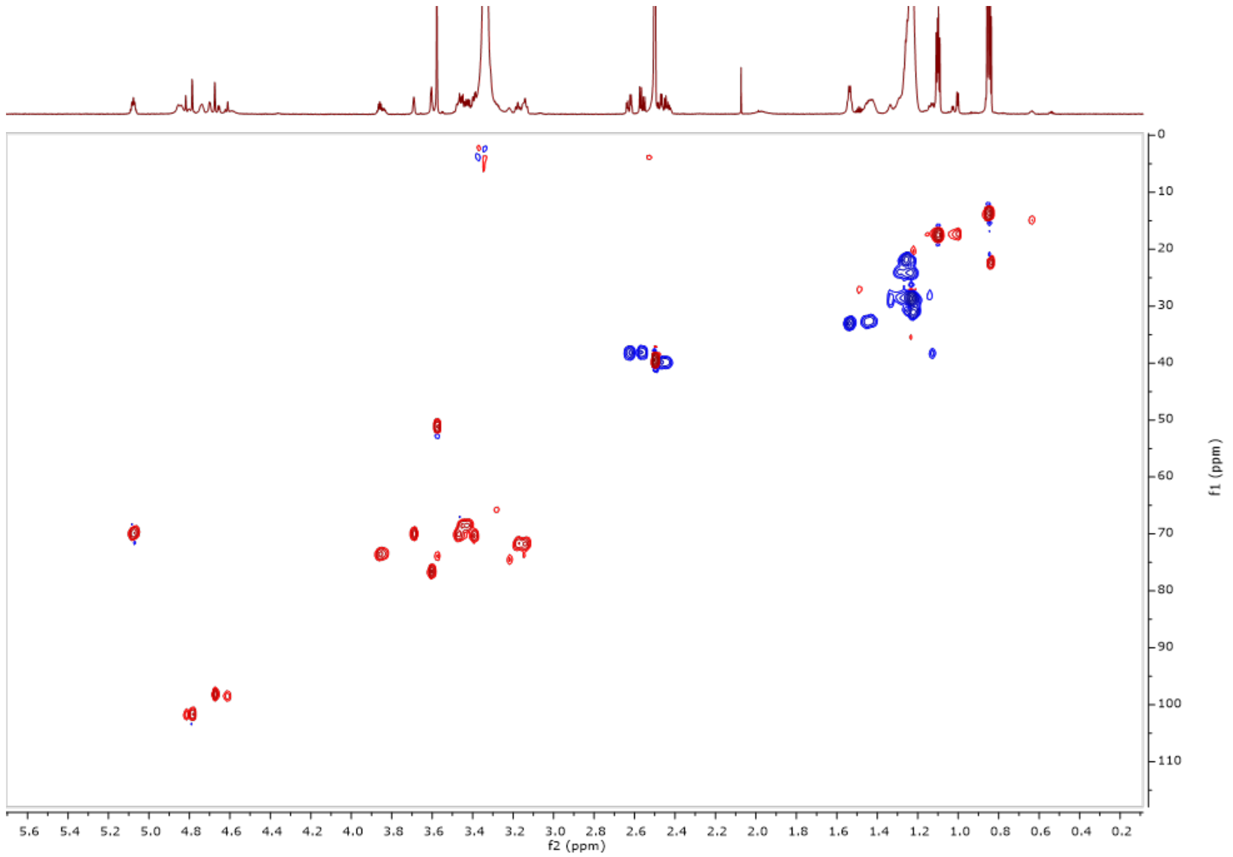

Figure S5. ^{1}H-^{13}C HSQC spectrum of rhamnolipid methyl ester, recorded in DMSO-d_6 at 900 MHz.

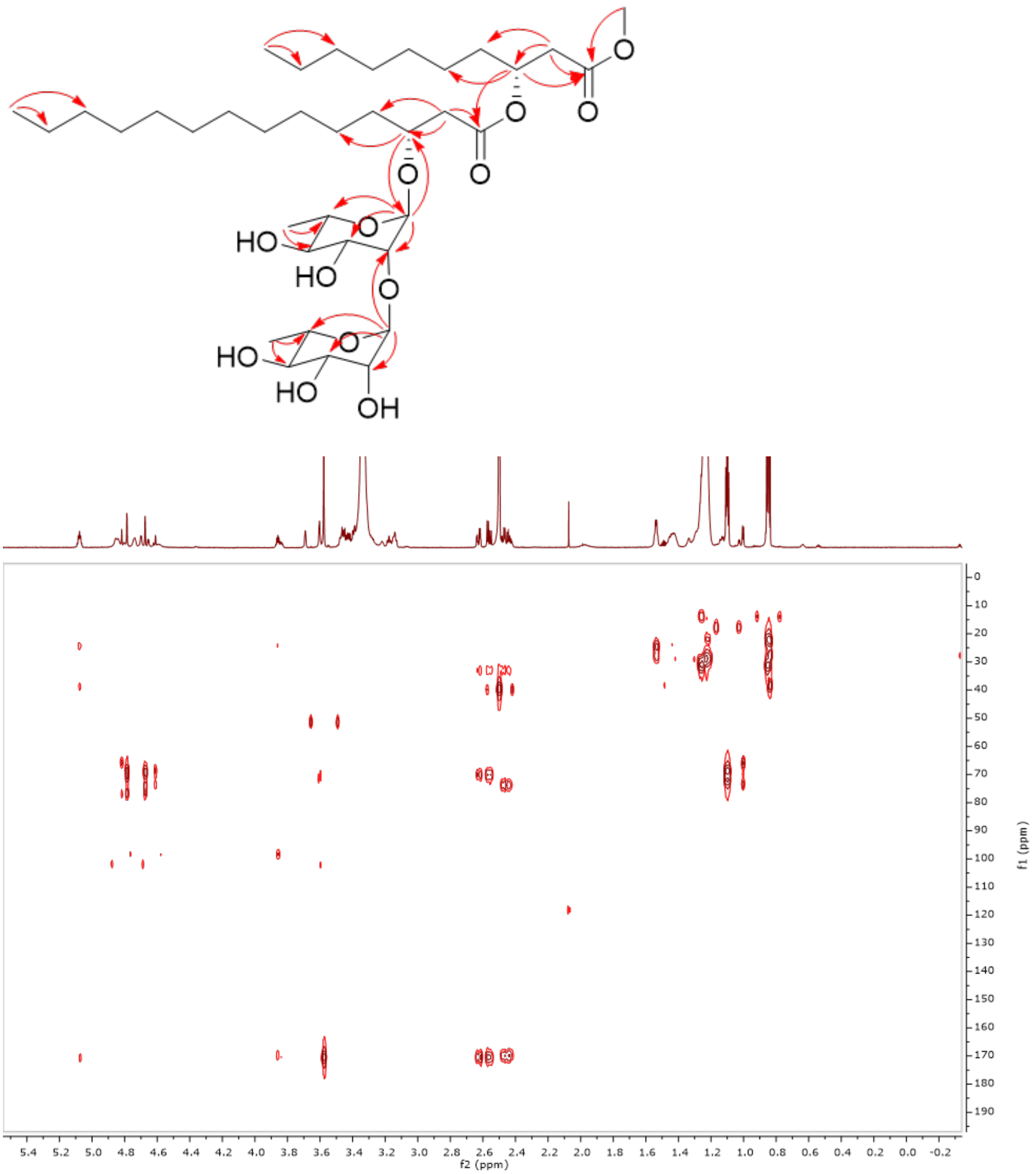

Figure S6. ^{1}H-^{13}C HMBC spectrum of rhamnolipid methyl ester, recorded in DMSO-d_6 at 900 MHz.

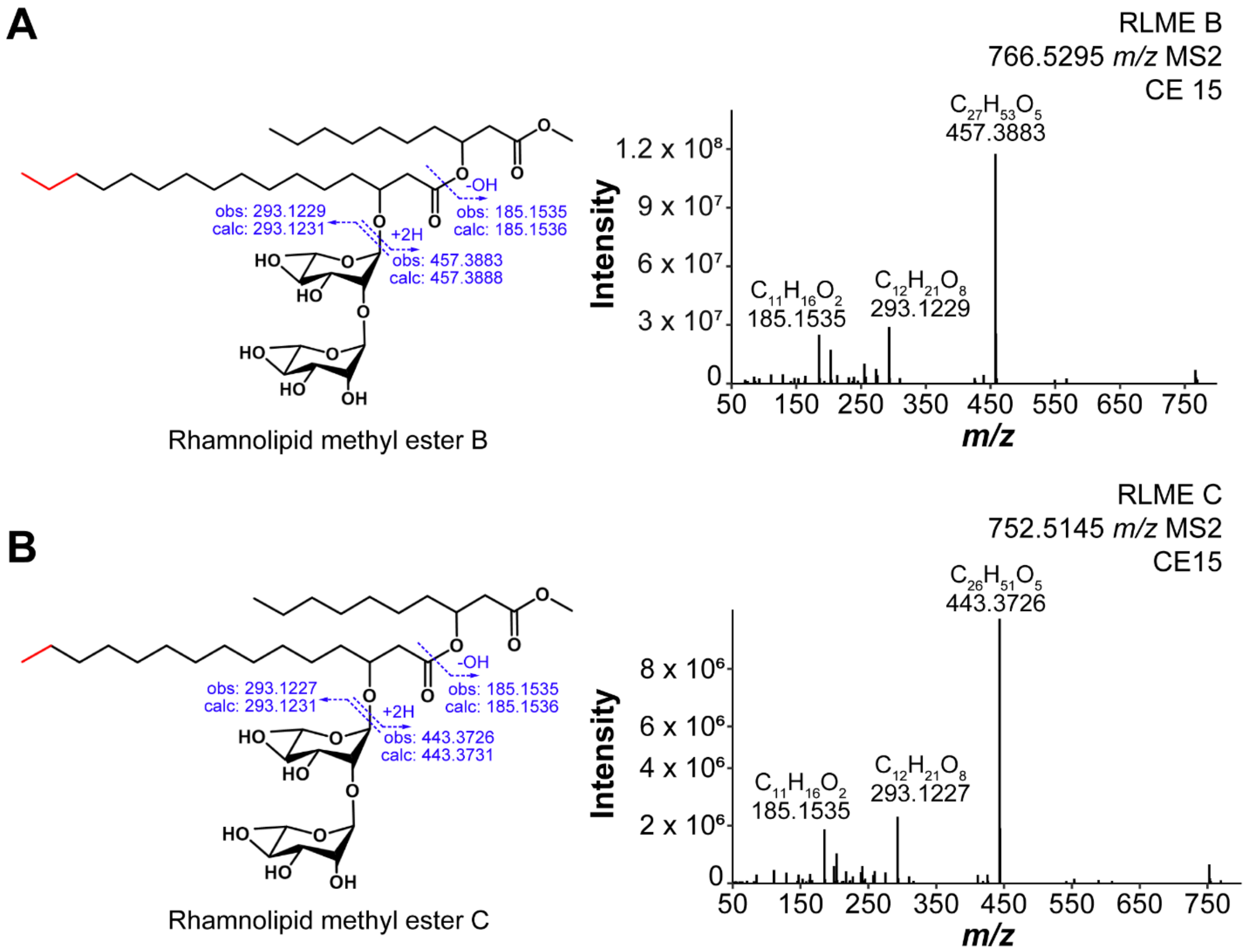

Figure S7. HRMS/MS spectra for rhamnolipid methyl ester B (A) and C (B). Dashed arrows indicate theoretical molecular ion fragments that produce *m/z* values observed (obs) in the experimental data shown on the right, along with theoretically calculated values (calc). HRMS/MS fragmentation spectra obtained using collision energy of 15 eV.

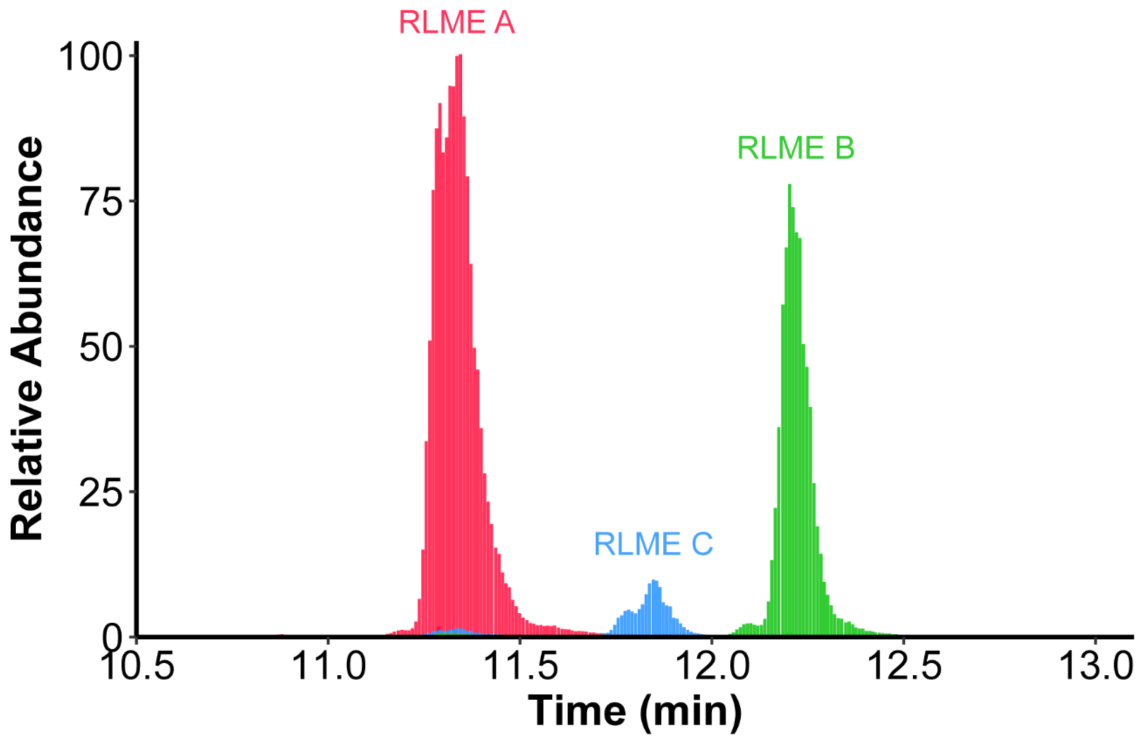

Figure S8. Relative abundance of rhamnolipid methyl ester A-C. A partially purified fraction from a *P. kirstenboschensis* F3 extract was analyzed using LC-HRMS.

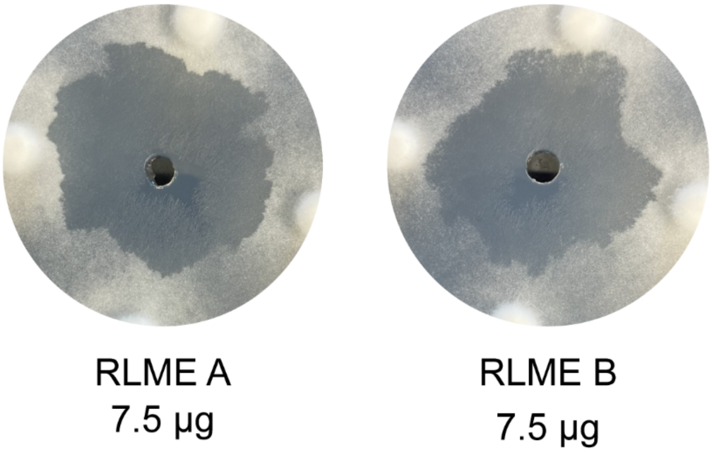

Figure S9. RLME A and B inhibition of *P. omphalodes* 1672. 15 µL of 0.5 mg/mL solutions of each compound were applied into the central well.

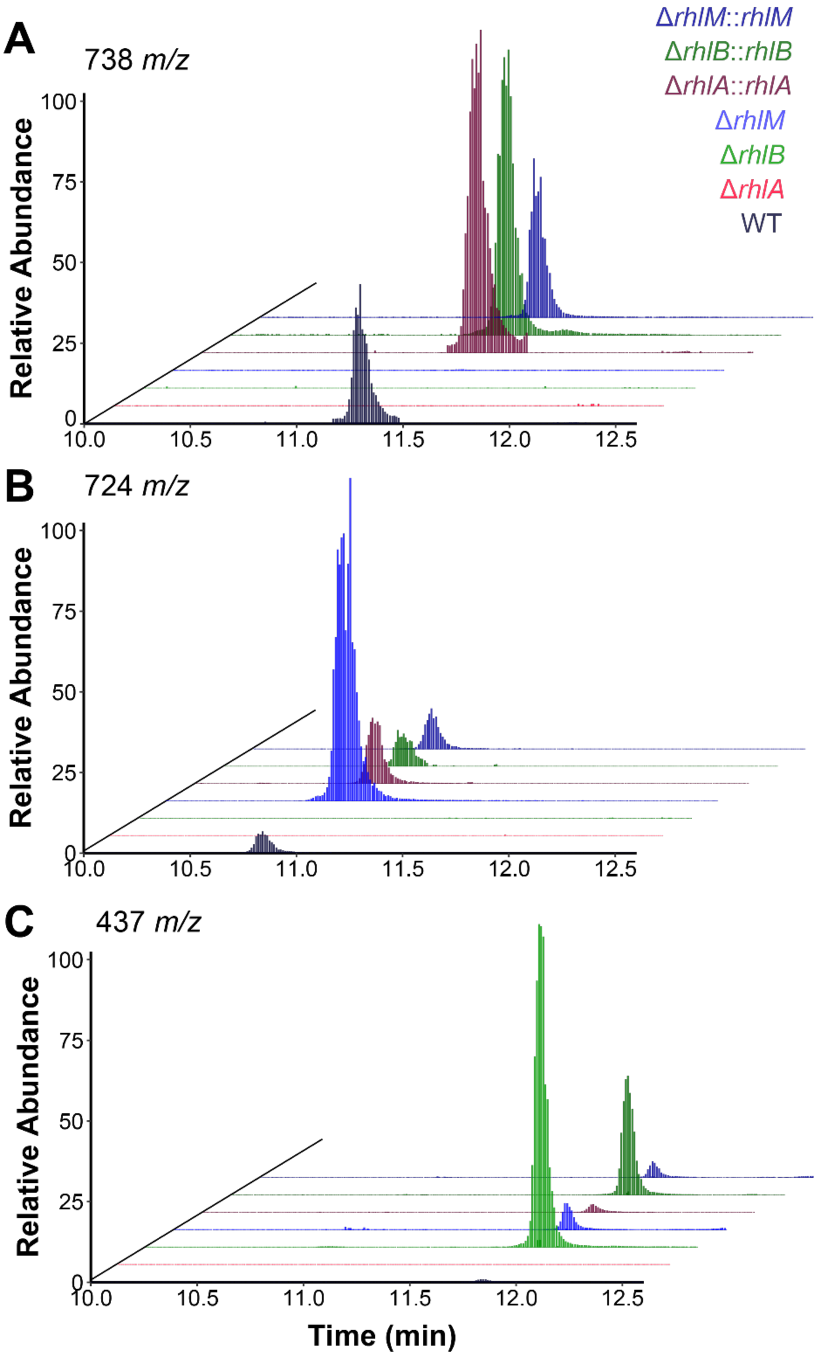

Figure S10. Extracted ion chromatograms (EIC) for *P. kirstenboschensis* F3 *rhl* mutants and genetic complement strains. (A) EIC for the RLME A ammonium adduct, 738 *m/z*, shows the absence of RLME A detected in three *rhl* knockout strains. RLME A production is rescued in genetic complementation strains. (B) EIC for the desmethyl RL ammonium adduct, 724 *m/z*, shows the accumulation of this intermediate in Δ*rhlM*, with low abundance detected in WT and complementation strains. (C) EIC for the HAA ammonium adduct, 437 *m/z*, shows the accumulation of this intermediate in Δ*rhlB*, with low abundance detected in WT and complementation strains. For each ion, intensities were normalized to the highest intensity detected among all strains.

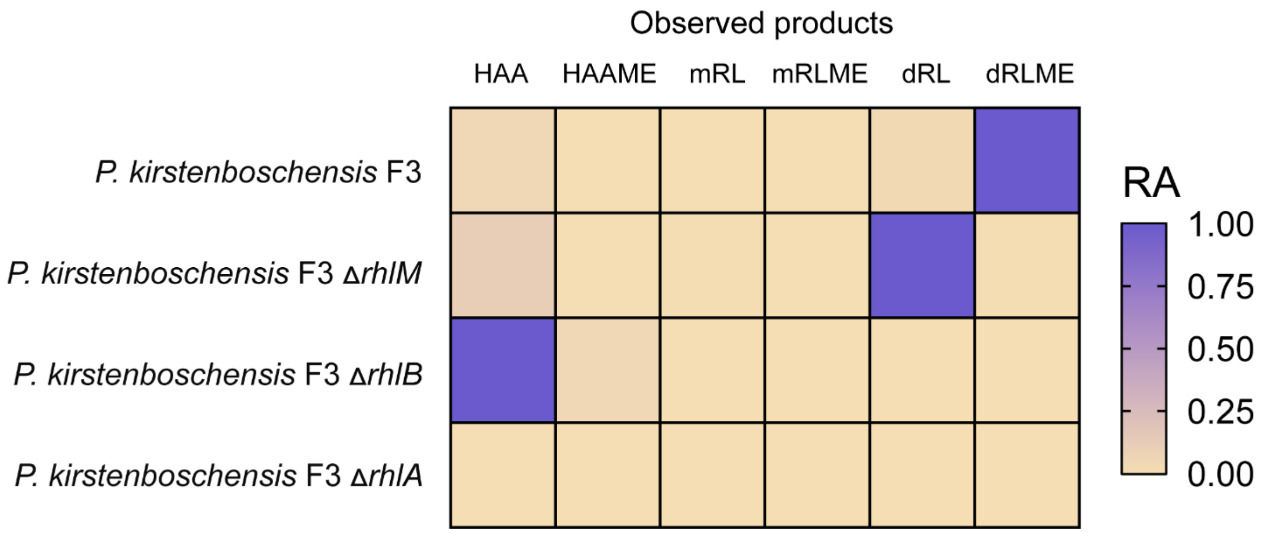

Figure S11. Heatmap of putative *rhl* biosynthetic intermediates. Production of RLME and possible *rhl* pathway biosynthetic intermediates of *P. kirstenboschensis* F3 wildtype and knockout mutants, observed using LC-MS. Relative abundance (RA) of ions found in *P. kirstenboschensis* F3 knockout strains and wild type (WT), by extracted ion chromatogram peak area and normalized by RA within each strain (slate blue, more abundant; tan, less abundant). HAA, 3-(3-hydroxyalkanoyloxy)alkanoate; HAAME, 3-(3-hydroxyalkanoyloxy)alkanoate methyl ester; mRL, mono-rhamnolipid; mRLME, monorhamnolipid methyl ester; dRL, di-rhamnolipid; dRLME, dirhamnolipid methyl ester.

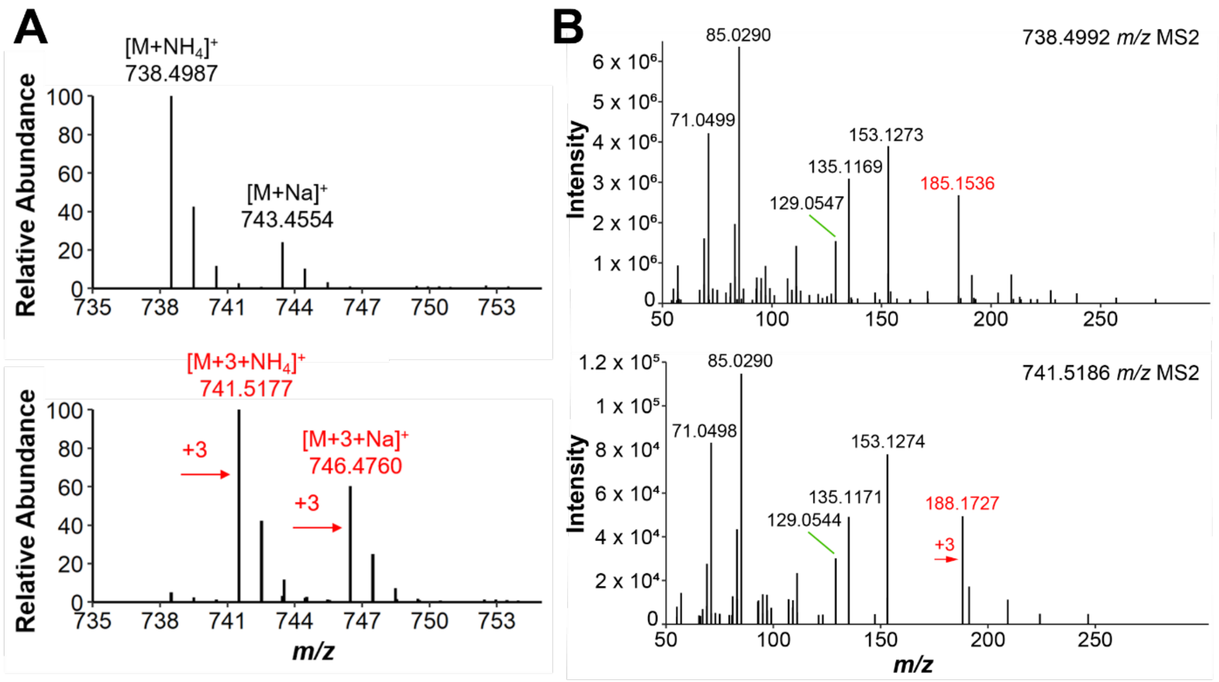

Figure S12. (A) Stable isotope labeling using D_3-Methionine verifies the biological origin of the carboxymethyl group, suggesting SAM-mediated methyltransferase activity. The +3 major isotopologues were observed for both ammoniated and sodiated adducts of RLME A in the labeled sample (bottom) compared to the unlabeled sample (top). (B) MS/MS spectra for the ammoniated adduct in the unlabeled sample (top) and labeled sample (bottom). Fragment 188.1537 *m/z*, corresponding to the acyl fragment containing the methyl ester, is the only fragment that shows incorporation of the D_3 label.

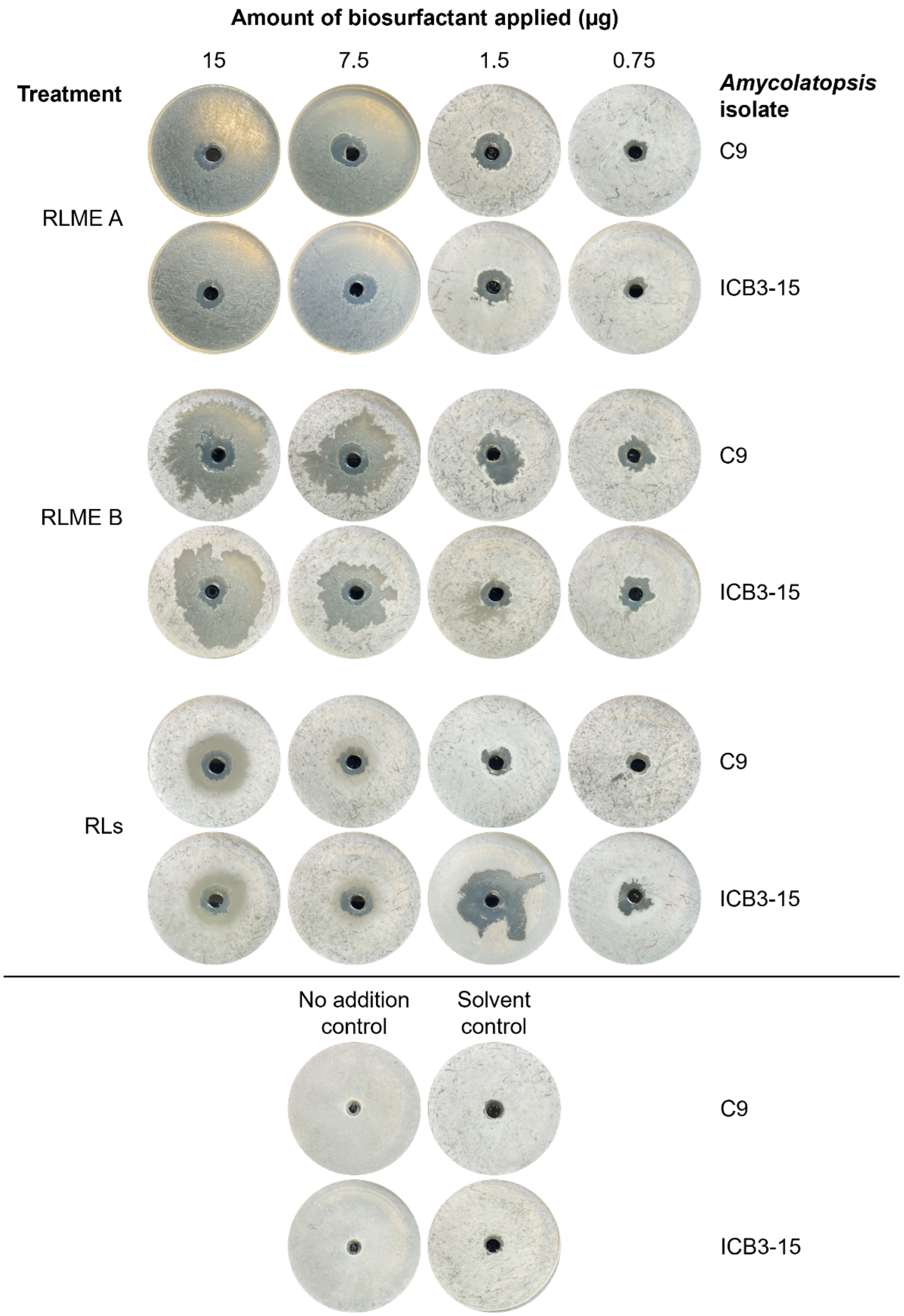

Figure S13. RLME A, RLME B, and RL inhibition of post-fire *Amycolatopsis* spp. isolates. Strain C9 was isolated from Blodgett Forest and strain ICB3-15 was isolated from the Illilouette Creek Basin Fire. RLMEs and RLs inhibit growth and suppress aerial hyphae development in *Amycolatopsis* spp. in a concentration-dependent manner.

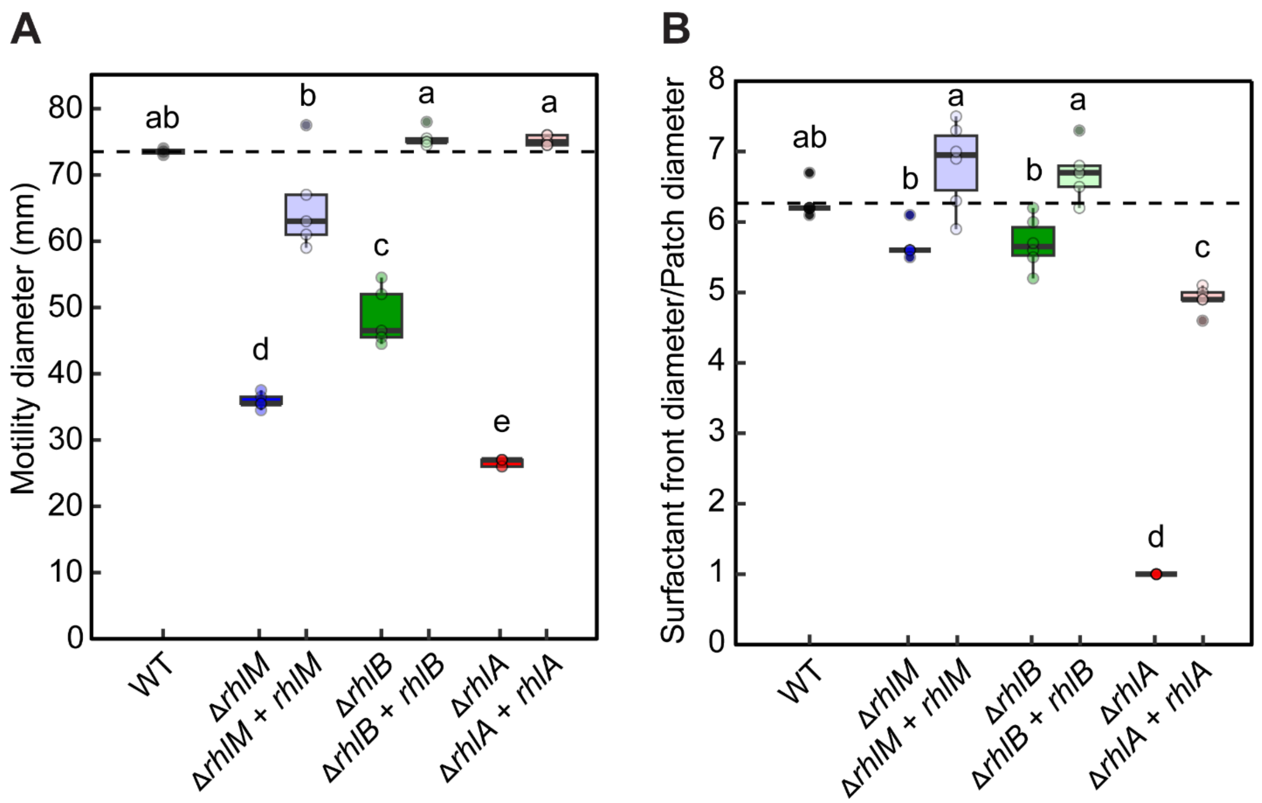

Figure S14. Quantification of swarming motility and surfactant fronts for *P. kirstenboschensis* F3 WT, *rhl* mutants, and genetic complementation strains. (A) Swarming diameters for *P. kirstenboschensis* strains. (B) Ratio of surfactant zone diameters to patch diameter for *P. kirstenboschensis* strains. Different letters indicate a statistically significant difference as determined by a one-way ANOVA and *post-hoc* Tukey's test ($p < 0.05$). Dashed line indicates mean wildtype measurement.

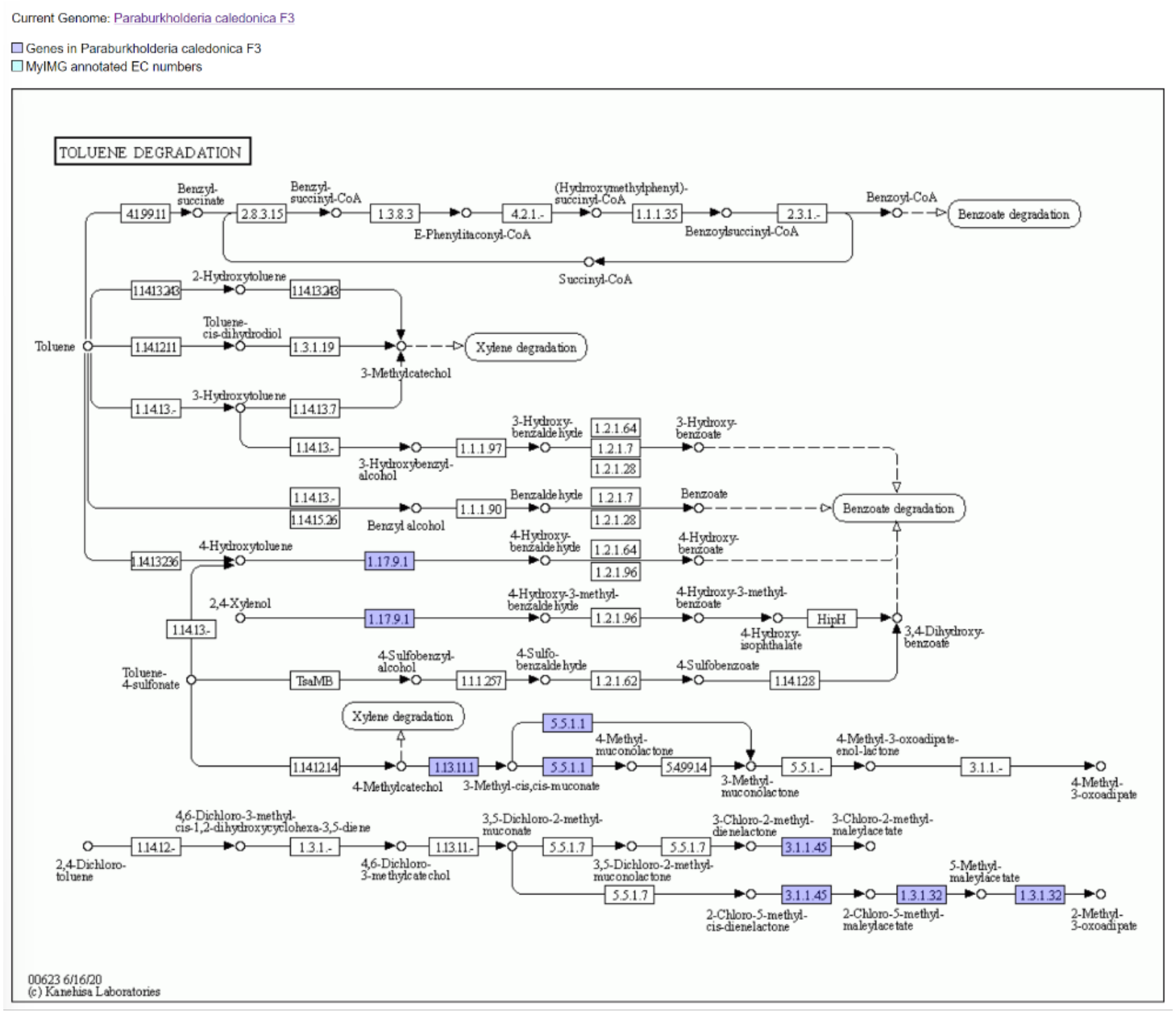

Figure S15. Predicted pathways for aromatic compound degradation in *P. kirstenboschensis* F3: toluene degradation pathways. KEGG analysis performed using JGI IMG-MER. Genes highlighted in lavender represent those found in the *P. kirstenboschensis* F3 genome.

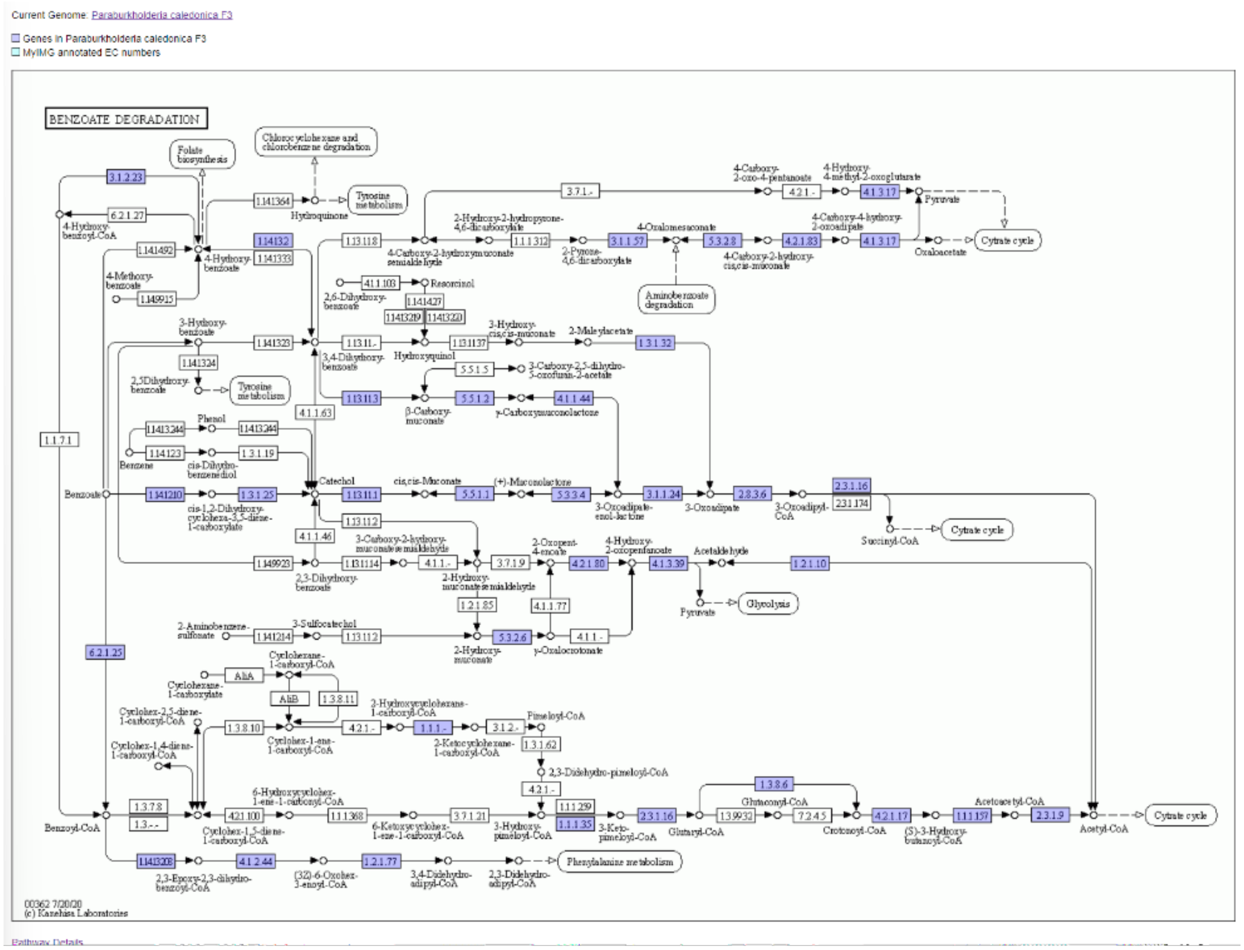

Figure S16. Putative pathways for aromatic compound degradation in *P. kirstenboschensis* F3: benzoate degradation pathways. KEGG analysis performed using JGI IMG-MER. Genes highlighted in lavender represent those found in the *P. kirstenboschensis* F3 genome.

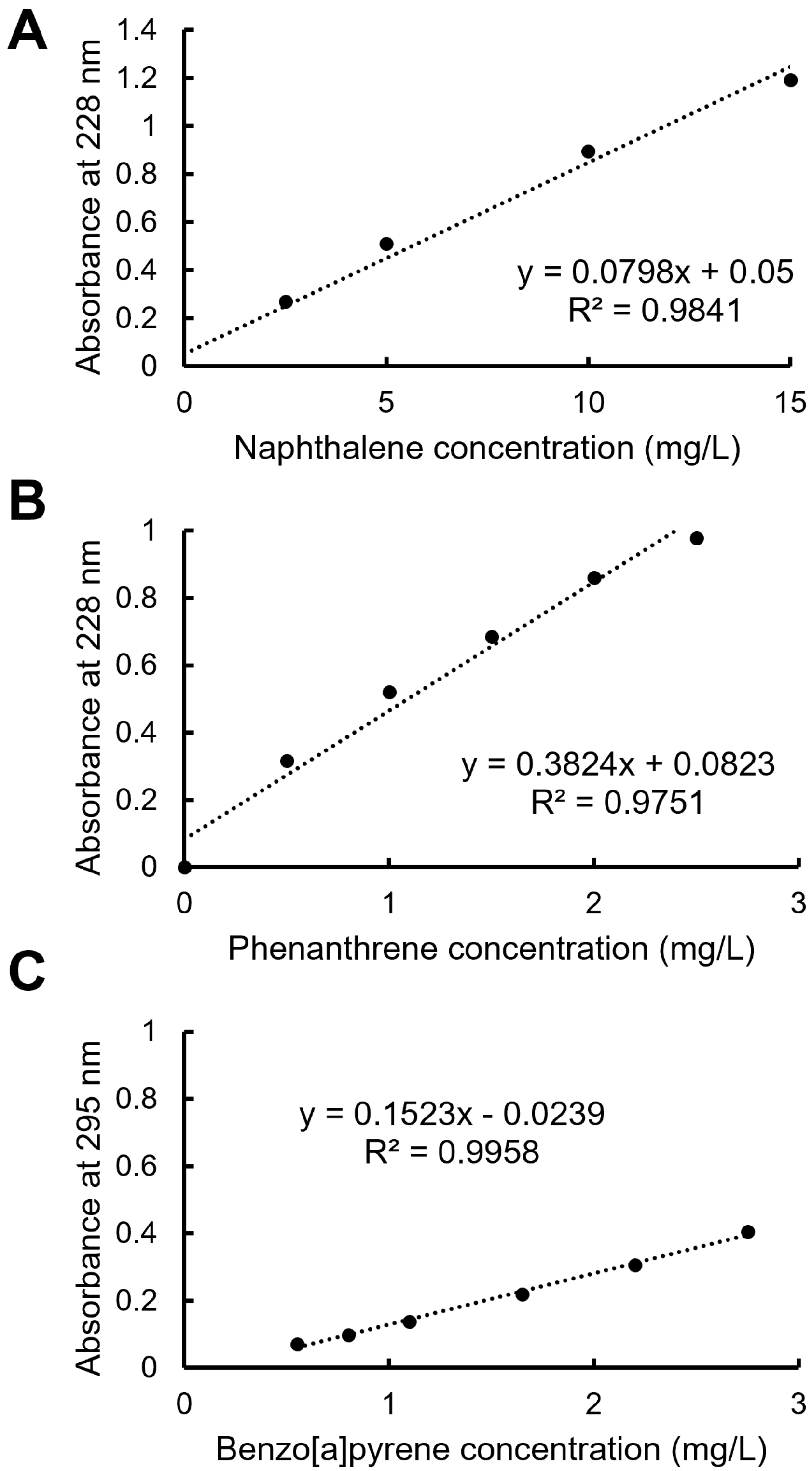

Figure S17. Standard curves for naphthalene (A), phenanthrene (B), and benzo[a]pyrene (C) used in PAH solubilization experiments.

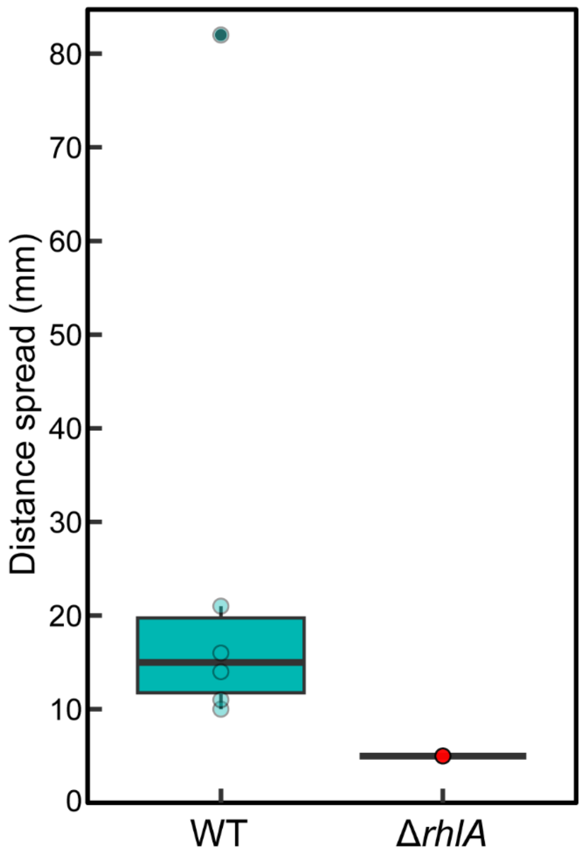

Figure S18. *P. kirstenboschensis* motility in race tube microcosms. Data represent six biological replicates for each strain, incubated in a race tube opposite of *Pyronema omphalodes* 1672.

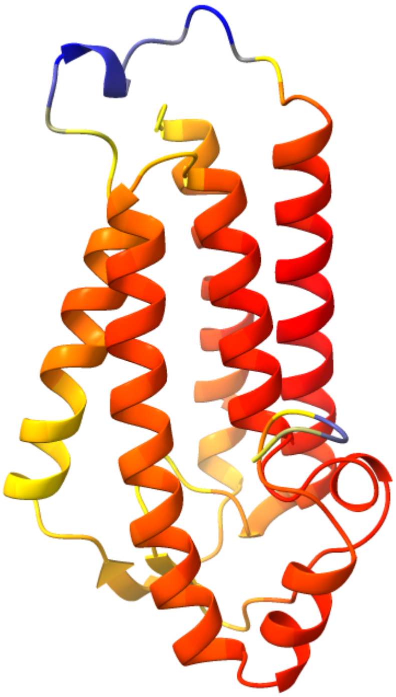

Figure S19. AlphaFold structure of RhlM colored by pLDDT score. Threshold values of 58.23, 77.75, and 98.11 were used for blue, yellow, and red, respectively.

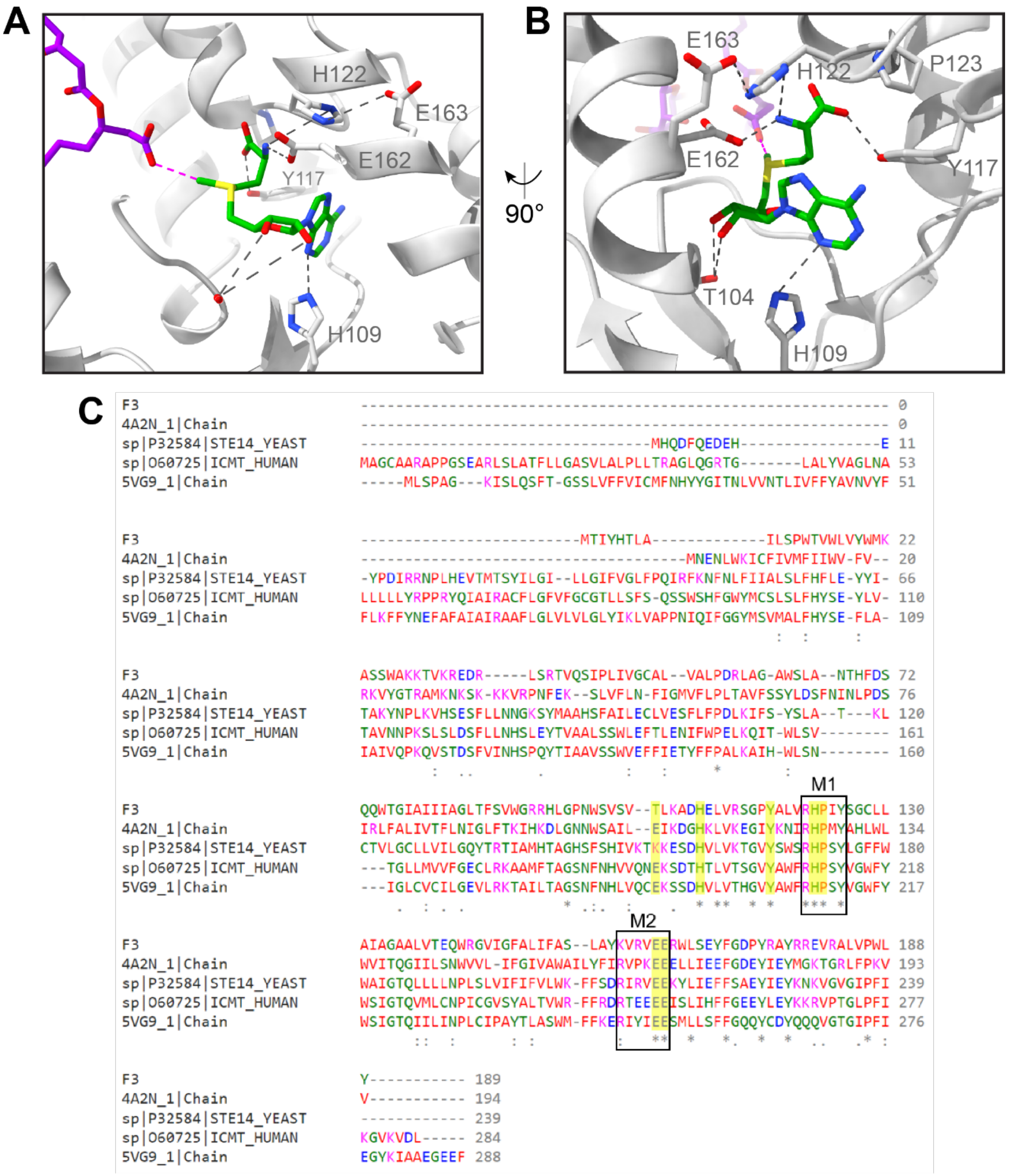

Figure S20. (A, B) Interactions with S-adenosyl methionine (SAM, green sticks). Putative interacting residues are shown as sticks. The cofactor is enclosed by conserved regions of RhlM. Gray dashed lines indicate hydrogen bonds. Magenta dashed line indicates measured distance between RL carboxylate O (methyl acceptor) and SAM methyl C (methyl donor). Atom coloring is: nitrogen, blue; oxygen, red; sulfur, yellow. (C) Multiple sequence alignment of RhlM (F3), Ma Mtase (4A2N), *Saccharomyces cerevisiae* ICMT (STE14_YEAST), *Homo Sapiens* ICMT (ICMT_HUMAN), and Tc ICMT (5VG9) made with Clustal Omega. M1 and M2 are conserved motifs within the cofactor-binding pocket of ICMT family proteins. Residues highlighted in yellow correspond to interacting residues shown in A and B.

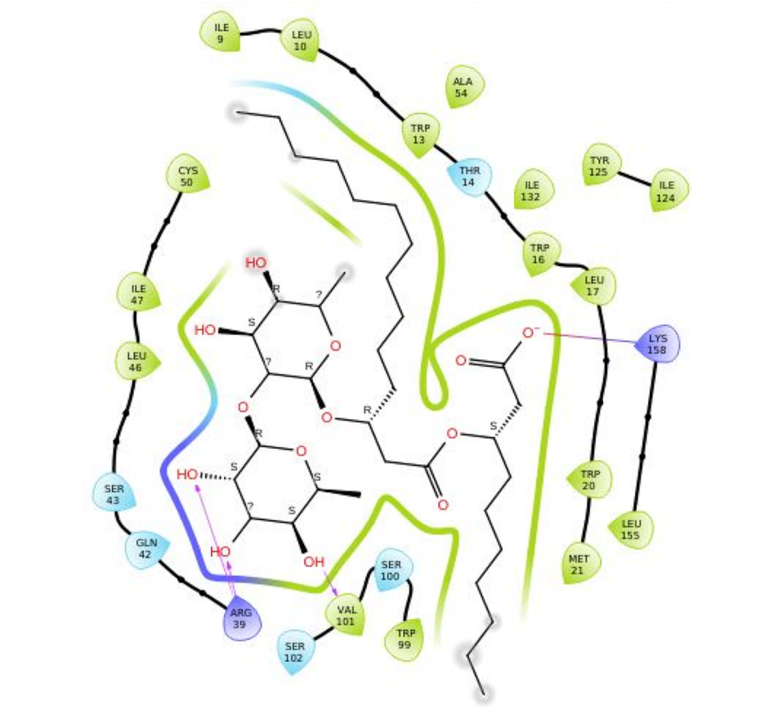

Figure S21. A 2D schematic ligand interaction diagram for RL and RhlM. Residues and the cartoon contact strip are colored by interaction type: green = hydrophobic, light blue = polar, dark blue = charged (positive). Atoms shaded with gray circles are solvent exposed.

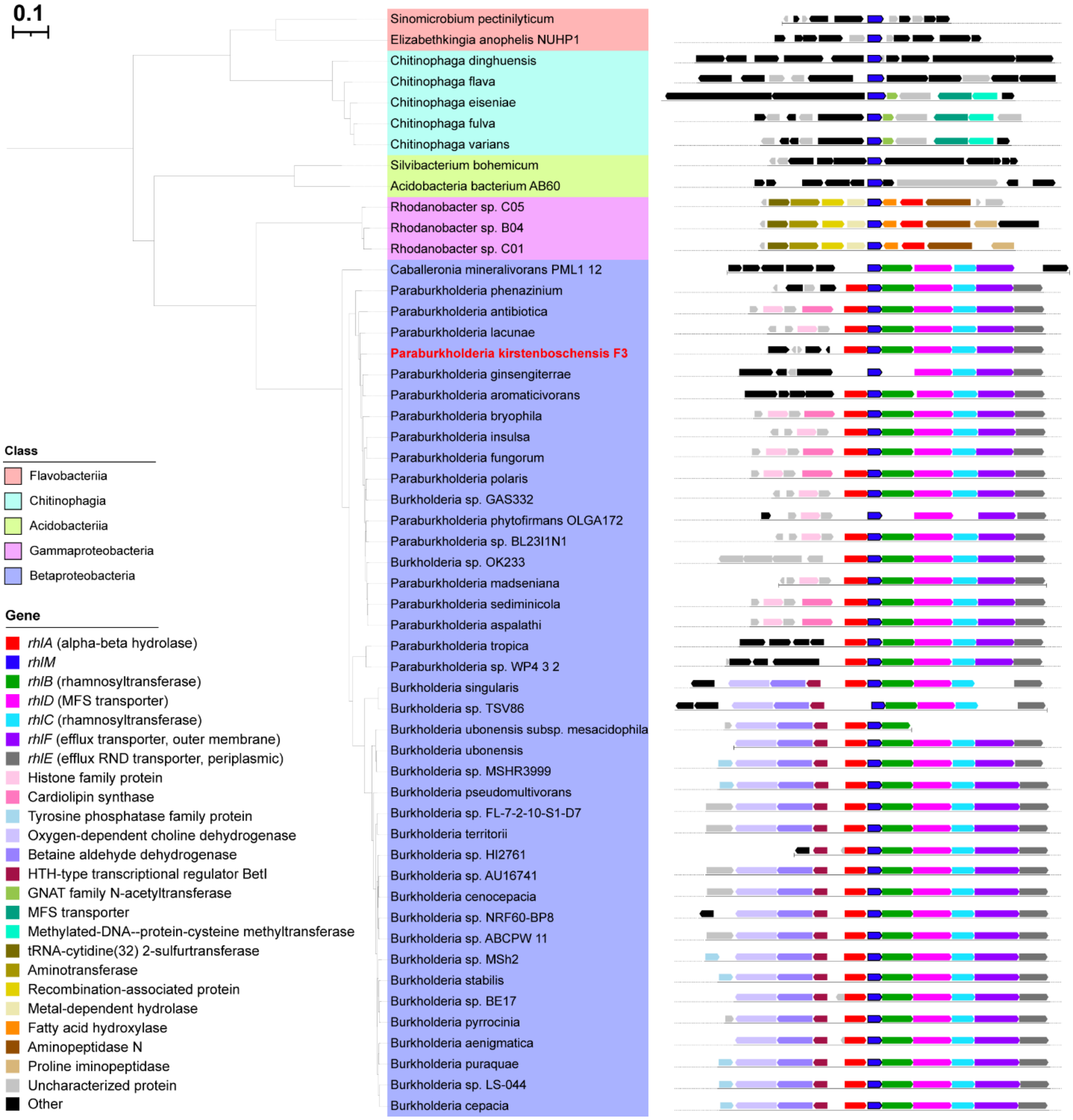

Figure S22. Species tree for strains in SSN Cluster 14 harboring RhlM homologs. The tree was constructed using KBase, and visualized and annotated in iTOL. The outgroup (*Methanosarcina acetivorans* C2A) was manually removed to facilitate visualization. Tree labels are colored by bacterial class. Genes are colored by annotated function in the EFI Genome Neighborhood Tool.
